# Xiaoyankangjun tablet alleviates dextran sulfate sodium-induced colitis in mice by regulating gut microbiota and JAK2/STAT3 pathway

**DOI:** 10.1007/s13659-024-00468-6

**Published:** 2024-08-12

**Authors:** Suqin Yang, Jingtao Huang, Wenjing Tan, Xiankun Xia, Dali Gan, Yalei Ren, Hanwen Su, Meixian Xiang

**Affiliations:** 1 School of Pharmaceutical Sciences, South-Central Minzu University, Wuhan, 430074 Hubei People’s Republic of China; 2 Department of Clinical Laboratory, Renmin Hospital of Wuhan University, Wuhan, 430060 Hubei People’s Republic of China

**Keywords:** Xiaoyankangjun tablet, Colitis, Nrf2/HO-1 pathways and JAK2/STAT3 pathways, Tight junction proteins, Gut microbiota, GPR43/41 receptor

## Abstract

Xiaoyankangjun tablet (XYKJP) is a traditional Chinese medicine formulation used to treat intestinal disorders in clinical practice. However, the specific therapeutic mechanism of action of XYKJP in colitis has not yet been elucidated. This study aimed to reveal the multifaceted mechanisms of action of XYKJP in treating colitis. The model established based on DSS-induced colitis in C57BL/6 mice was employed to estimate the effect of XYKJP on colitis, which was then followed by histological assessment, 16S rRNA sequencing, RT-qPCR, ELISA, and Western blot. XYKJP alleviated the symptoms of DSS-induced colitis mainly by reducing oxidative stress, inflammatory responses, and intestinal mucosal repair in colitis tissues. In addition, XYKJP regulated the intestinal flora by increasing the relative abundance of *Akkermansia* and *Bifidobacterium* and reducing the relative abundance of *Coriobacteriaceae_UCG-002*. Mechanistically, XYKJP increased the content of short-chain fatty acids (SCFAs) in the feces, particularly propanoic acid and butyric acid, activated their specific receptor GPR43/41, furthermore activated the Nrf2/HO-1 pathway, and suppressed the JAK2/STAT3 pathway. XYKJP significantly alleviated the symptoms of experimental colitis and functioned synergistically by regulating the intestinal flora, increasing the production of SCFAs, and activating their specific receptors, thereby repressing oxidative stress and inflammation.

**Graphical Abstract:**

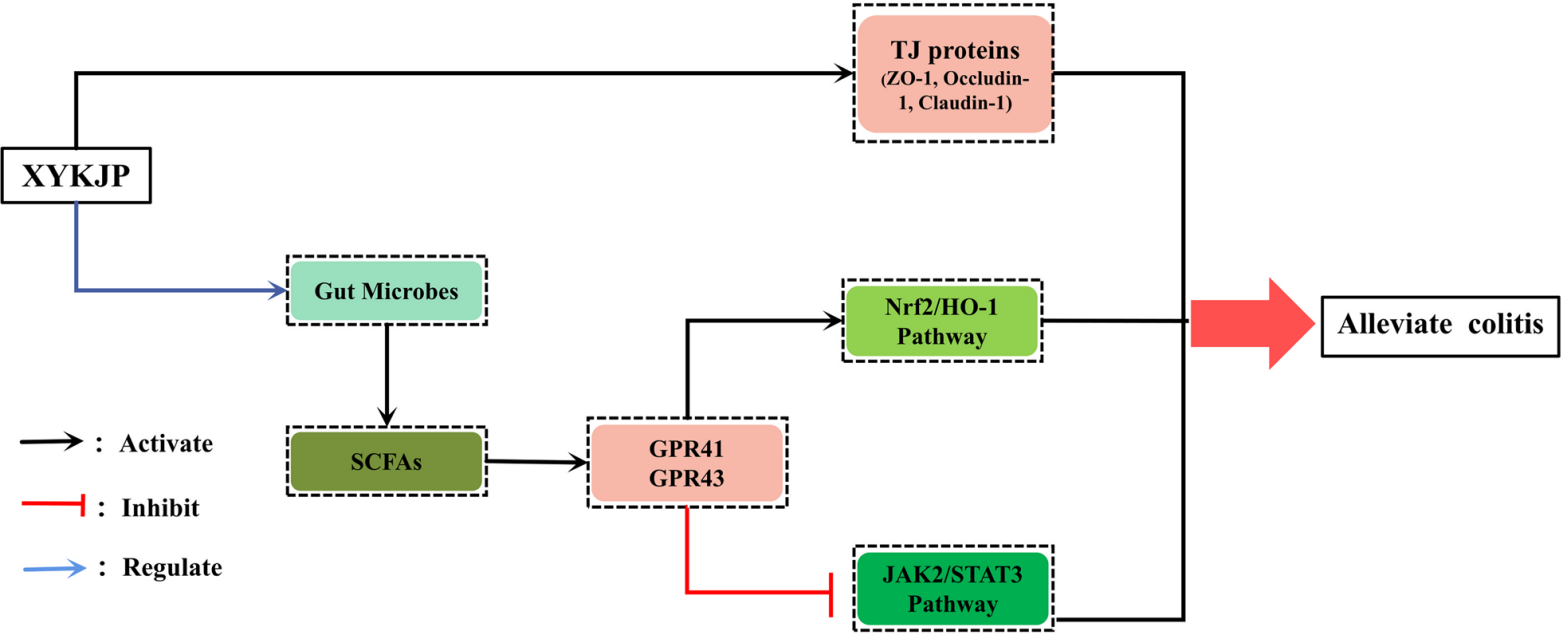

**Supplementary Information:**

The online version contains supplementary material available at 10.1007/s13659-024-00468-6.

## Introduction

Colitis, a challenging inflammatory disease of the gastrointestinal tract, presents with clinical symptoms such as pain, diarrhea, bloody stools, and weight loss [1]. The disease is triggered by a variety of pathogenic factors, including inheritance, immune dysfunction, intestinal barrier damage, and an imbalance in intestinal flora complexes [2]. The World Health Organization (WHO) classifies colitis as a modern, stubborn disease due to the difficulty in achieving full recovery and the associated risk of cancer [3]. Current clinical drugs used for colitis, such as glucocorticoids, 5-aminosalicylic acid, and immunosuppressants, have limitations and come with a range of adverse side effects, including allergic reactions and intolerance [4]. Fecal microbial transplantation (FMT) has shown promise in improving colitis symptoms by regulating flora imbalance; however, various aspects of donor selection and regimen strategies associated with FMT need to be evaluated [5]. Therefore, the search for novel drugs with balanced efficacy and biosafety is a critical need, providing reassurance about the future of colitis treatment.

The Xiaoyankangjun tablet (XYKJP) is a compound preparation for Traditional Chinese medicine (TCM) (pharmaceutical batch number: Z20220026) obtained from the Renmin Hospital of Wuhan University. XYKJP is composed of *Scutellaria baicalensis* *Georgi.* (Huangqin), *Sanguisorba officinalis L*. (Diyu), and *Punica granatum L.* (Shiliupi). The XYKJP formula, a mainstay at Renmin Hospital of Wuhan University for over 30 years, has consistently shown excellent therapeutic effects against bacillary dysentery and intestinal disorders. Its key components, baicalin, ellagic acid, and gallic acid, are distinguished by their potent anti-inflammatory and antioxidant effects, providing a clear understanding of its mechanisms of action [6–8].

As one of the main herbs in XYKJP prescriptions, Huang Qin has been commonly used for thousands of years for its various protective effects against liver and intestinal disorders [9]. For instance, Huang Qi decoction inhibits DSS-induced colitis inflammation in vivo by modifying the intestinal flora and amino acid synthesis, activating the mTOR pathway, and restoring intestinal barrier integrity [10]. The Huang Qin-derived homogeneous polysaccharide, named SP2-1, was effective in alleviating the symptoms of ulcerative colitis, regulating the population and abundance of intestinal flora, and ultimately restoring colonic function [11]. Diyu, a prominent traditional herbal medicine, is a beacon of hope in the treatment of tissue repair and inflammatory diseases, and in the prevention and treatment of tumors due to its potential immune-enhancing, hemostatic, and antibacterial activities [12]. In a DSS-induced colitis model, Diyu and its active ingredients protect against intestinal inflammation by inducing macrophage autophagy [13]. Shiliupi, the pericarp of pomegranate, is a medicinal plant with a wide range of distributions and applications [14]. For generations, shiliupi has been used to treat ulcers and diarrhea and has anti-inflammatory, anti-viral, and anti-fibrotic properties. Shiliupi has also been reported to inhibit colitis and improve intestinal flora, thus playing a key role in gastrointestinal disorders [15]. XYKJP is a composite of huangqin, diyu, and shiliupi, with robust synergistic modulation of anti-inflammatory and flora regulatory functions, and deserves further investigation.

To date, the potential of XYKJP in combating colitis has not been fully investigated in previous studies. Accordingly, this study was designed to elucidate the roles and mechanisms of XYKJP in the treatment of colitis. We evaluated the protective role of XYKJP against DSS-induced colitis using a mouse model. In this study, we found that XYKJP plays a critical role in reversing intestinal dysbiosis, a condition characterized by an imbalance in the gut microbiota, in colitis. We also detected oxidative stress-related biomarkers, inflammatory cytokines, and potential nuclear factor erythroid 2-related factor 2 (Nrf2)/heme oxygenase 1 (HO-1) and janus kinase 2 (JAK2)/signal transducer and activator of transcription 3 (STAT3) pathways in the colitis tissues. Our study contributes to deciphering the novel mechanisms of XYKJP in colitis and the development of novel therapeutic strategies.

## Results

### LC–MS analysis of XYKJP components

**Fig. 1:**
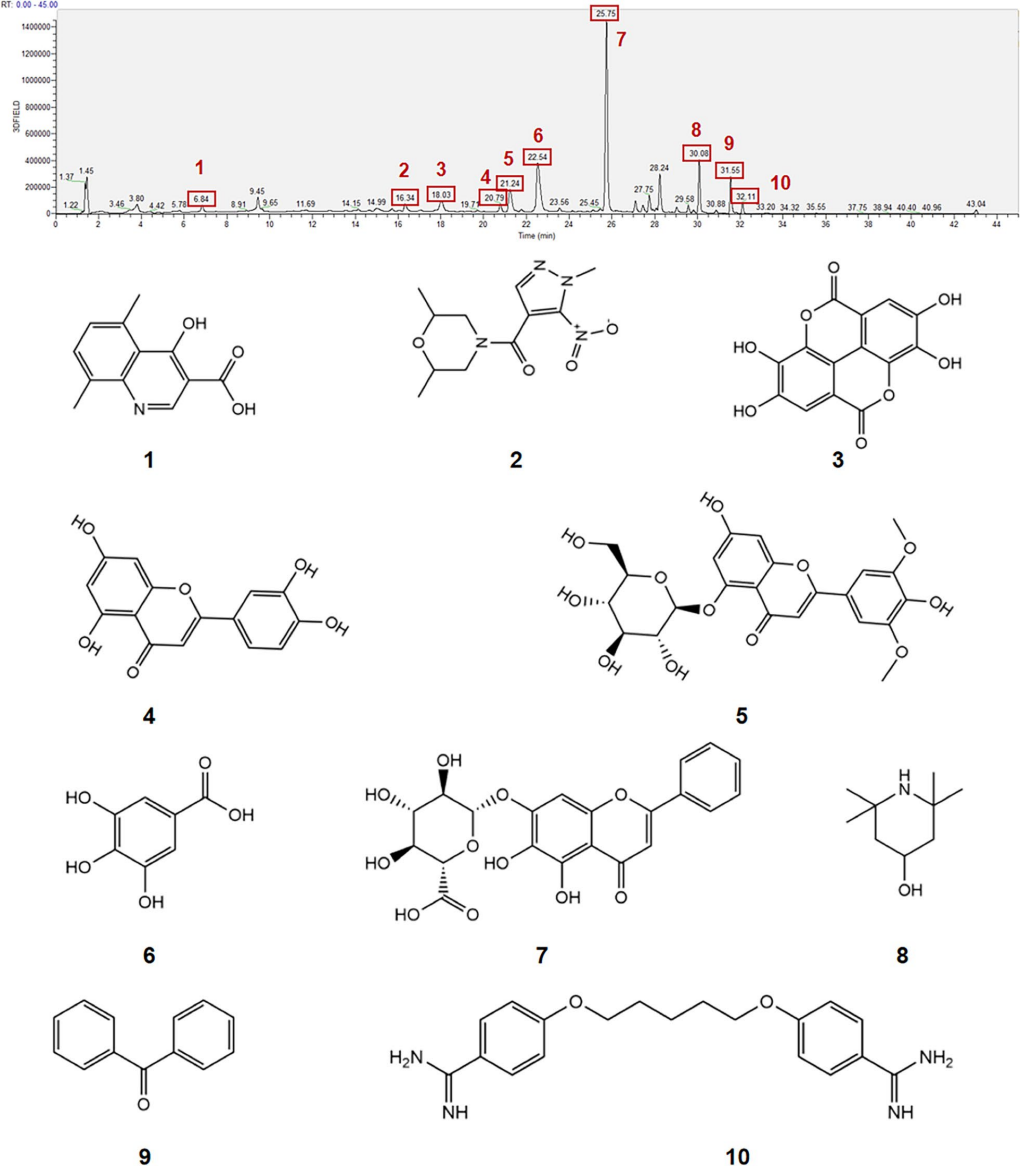
Analysis of active components in XYKJP by LC–MS. Liquid chromatogram: The chromatographic peak of the standard of the sample. Peak 1: 4-hydroxy-5,8-dimethyl quinoline-3-carboxylic acid; Peak 2: (2,6-dimethyl morpholino) (1-methyl-5-nitro-1H-pyrazol-4-yl) methanone; Peak 3: Ellagic acid; Peak 4: Luteolin; Peak 5: Tricin 5-O-β-D-glucoside; Peak 6: Gallic acid; Peak 7: Baicalin; Peak 8: 2,2,6,6-Tetramethyl-4-piperidinol; Peak 9: Benzophenone; Peak 10: Pentamidine

To obtain information about the compound composition of XYKJP, the methanolic extract of XYKJP was analyzed by LC–MS, and 10 compounds were identified (Fig. 1). Among them, baicalin, gallic acid, and ellagic acid were identified as the quality markers of *Scutellaria baicalensis*, *Dictyostelium*, and *Punica granatum*, respectively, according to the rigorous standards of the Chinese Pharmacopoeia. The contents of baicalin (35.40 mg/g), gallic acid (6.93 mg/g), and ellagic acid (21.20 mg/g) are high in comparison and are the main active substances that exert medicinal effects.

### XYKJP relieved the symptoms of colitis in mice

**Fig. 2:**
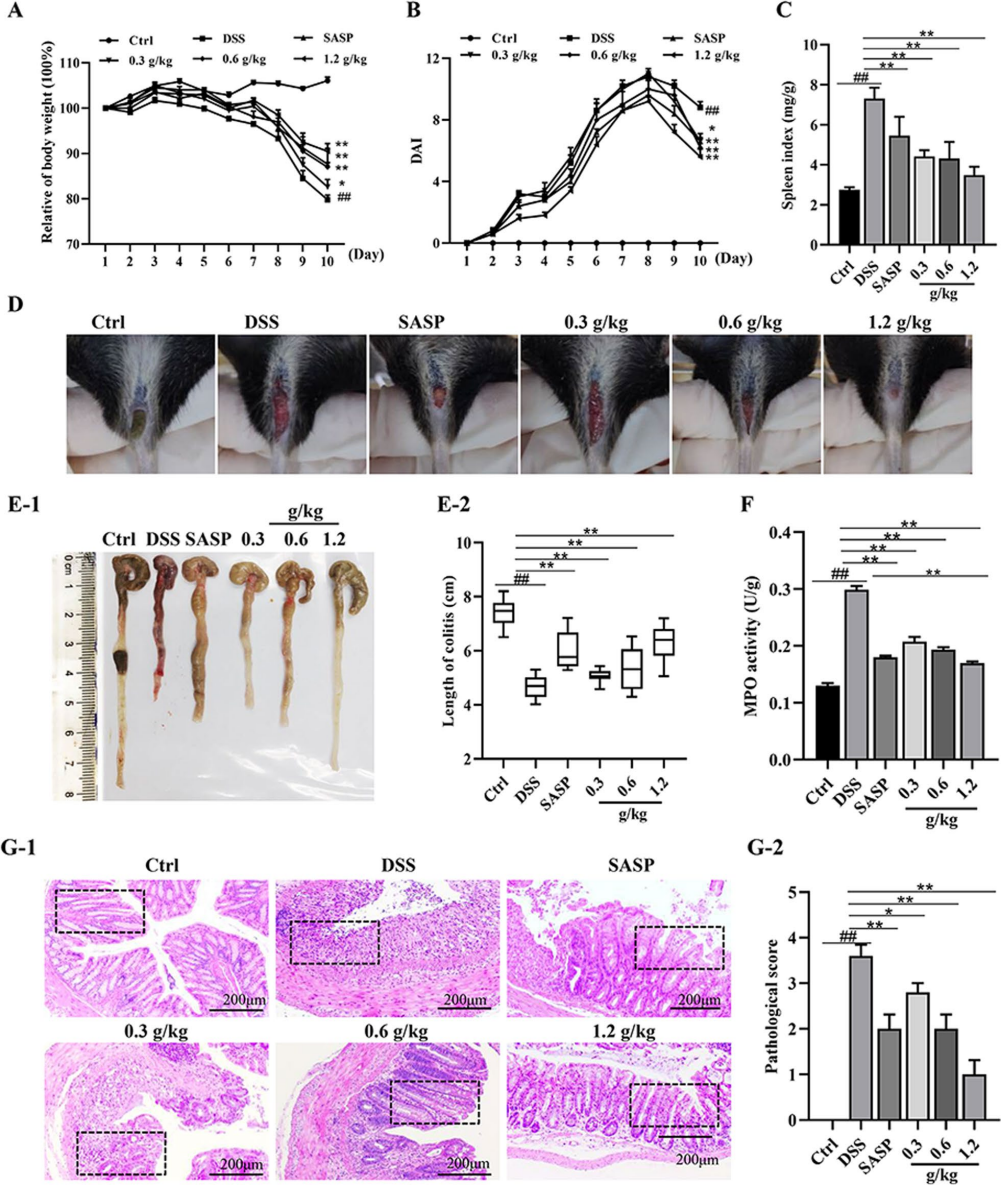
XYKJP improved the pathological symptoms of colitis mice. **A** Each group's mice body weight changed daily within 10 days. **B** The result of the DAI score was estimated daily. **C** The result of the spleen index was calculated. **D** Perianal blood stains were observed and photographed. **E-1**, **E-2** The result of each group's mice colon length was measured and then calculated. **F** The result of the MPO activity of colon tissues. **G-1**, **G-2** The result of representative H&E images of the colon (scale bars = 200 μm) were captured to obtain the pathological evaluation. Data were expressed as ± SEM (n = 10), *P < 0.05, **P < 0.01, ***P < 0.001

Next, we explored the potential of XYKJP to alleviate colitis symptoms in vivo. We observed a dose-dependent reduction in weight loss, disease activity index (DAI) score, and splenic index in the XYKJP group compared to the DSS group (Fig. 2A–C). Stool blood levels also showed significant improvement with XYKJP treatment (Fig. 2D). Furthermore, XYKJP treatment significantly alleviated DSS-induced colonic shortening (Fig. 2E1–E2). Myeloperoxidase (MPO) activity was significantly reduced by XYKJP treatment, indicating a suppression of inflammatory symptoms in DSS-treated mice (Fig. 2F). Importantly, hematoxylin and eosin (H&E) staining revealed a reversal of structural damage to the colonic mucosa, dislodgment of thrush cells, severe inflammatory cell infiltration, and edema in the mucosal and submucosal layers of the DSS group (Fig. 2G1–G2). XYKJP treatment effectively reversed these effects in a dose-dependent manner, raising intriguing questions about its potential to significantly improve the pathological symptoms of colitis.

### XYKJP alleviated gut dysbiosis of colitis in mice

**Fig. 3:**
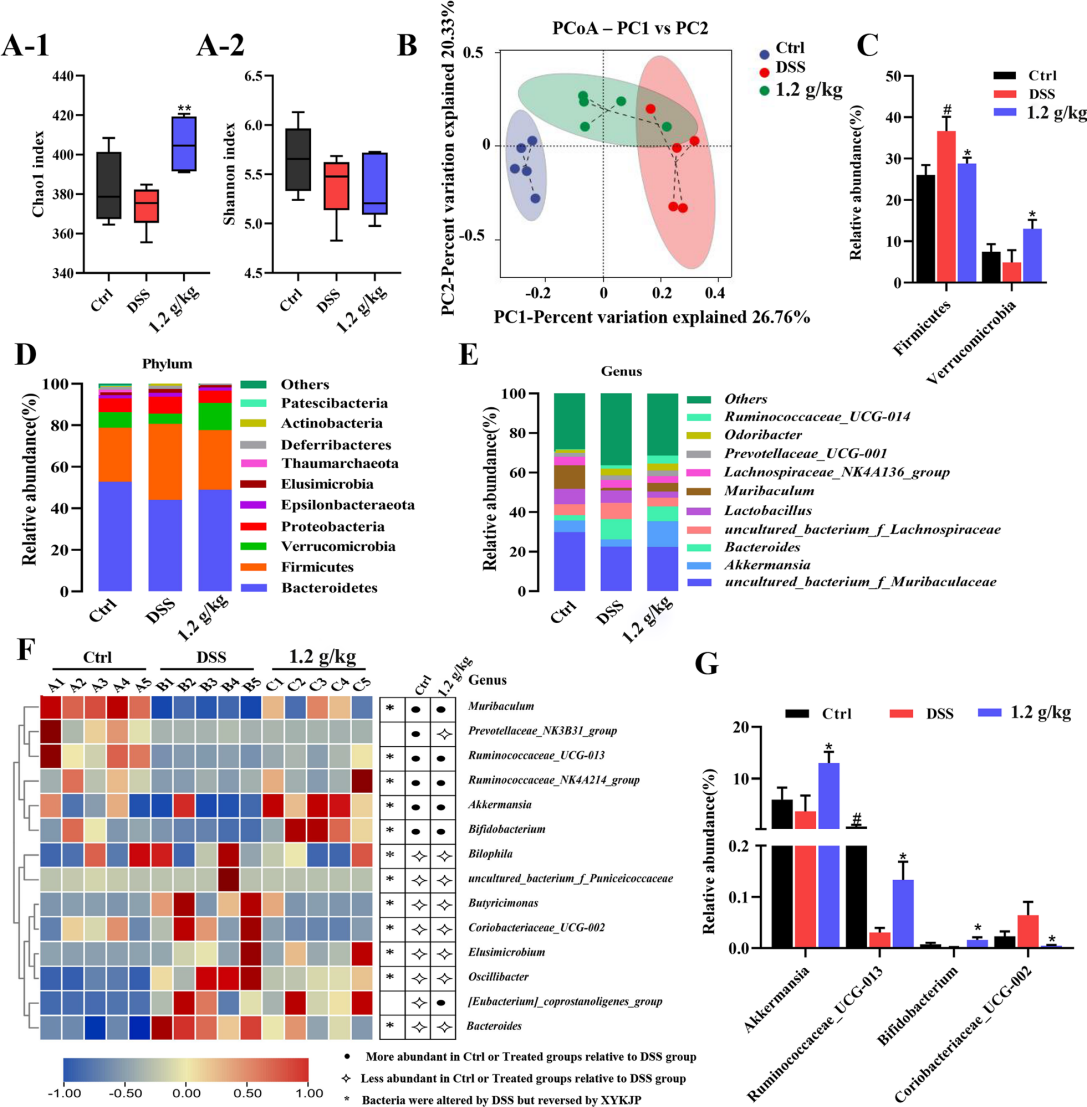
XYKJP alleviated the dysbiosis of the intestinal flora of DSS-induced colitis mice. **A-1** The result of the Chao index. **A-2** The result of the Shannon index. **B** Principal Coordinates Analysis (PCoA) for β-diversity. **C** The result of the relative abundance of two dominant bacteria. **D** Differences of gut microbes at the phylum level. **E** Differences of gut microbial at the genus of level. **F** The heat map at the genus level. **G** The result of the relative abundance of four dominant bacteria. Data were expressed as ± SEM (n = 6), *P < 0.05, **P < 0.01, ***P < 0.001

The intestinal microbiota, a key player in maintaining intestinal homeostasis, has been a subject of significant research. The reduced diversity and altered composition of the intestinal flora in patients with colitis are crucial pathological features [16]. Our study, which found that the DSS group had lower Chao and Shannon indices than the control group, and that XYKJP treatment resulted in an upward trend in the Chao index (Fig. 3A), contributes to this body of knowledge.

The β-diversity was assayed by using principal coordinates analysis (PCoA), which was based on the unweighted UniFrac statistical algorithm, indicating that the intestinal microbiota structure was significantly different from that of the Ctrl group, DSS group, and XYKJP group (Fig. 3B). The dominant bacteria were *Bacteroidetes*, *Firmicutes*, *Verrucomicrobia*, and *Proteobacteria* in XYKJP groups. *Firmicutes* and *Proteobacteria* abundances in the DSS group increased; however, the abundance of *Verrucomicrobia* decreased (Fig. 3C). XYKJP treatment markedly decreased *Firmicutes* abundance and restored *Verrucomicrobia* abundance (Fig. 3D).

At the genus level, XYKJP treatment was found to have a significant positive impact. It increased *Coriobacteriaceae* UCG-002 abundance (Fig. 3E) and beneficial bacteria, including *Akkermansia*, *Bifidobacterium*, and *Ruminococcaceae*_UCG-013 (Fig. 3G). In essence, XYKJP induced a relative abundance of beneficial bacteria and decreased harmful bacteria, offering hope for improving intestinal microbiota dysregulation in mice with colitis.

Our study used LEfSe analysis to identify statistically significant biomarkers in the different groups and to identify the dominant microorganisms. The numbers of specific taxa in the control, DSS, and XYKJP groups were 3, 10, and 11, respectively (Fig. S1B). Linear discriminant analysis (LDA) was carried out to distinguish bacterial taxa with remarkable differences (LDA score ≥ 4). The results showed that XYKJP, by restoring the balance of the intestinal microbiota, partially ameliorated the intestinal microbiota imbalance in mice with colitis (Fig. S1).

### XYKJP treatment promoted the production of short-chain fatty acids (SCFAs) and activated GPR41/43 proteins

**Fig. 4:**
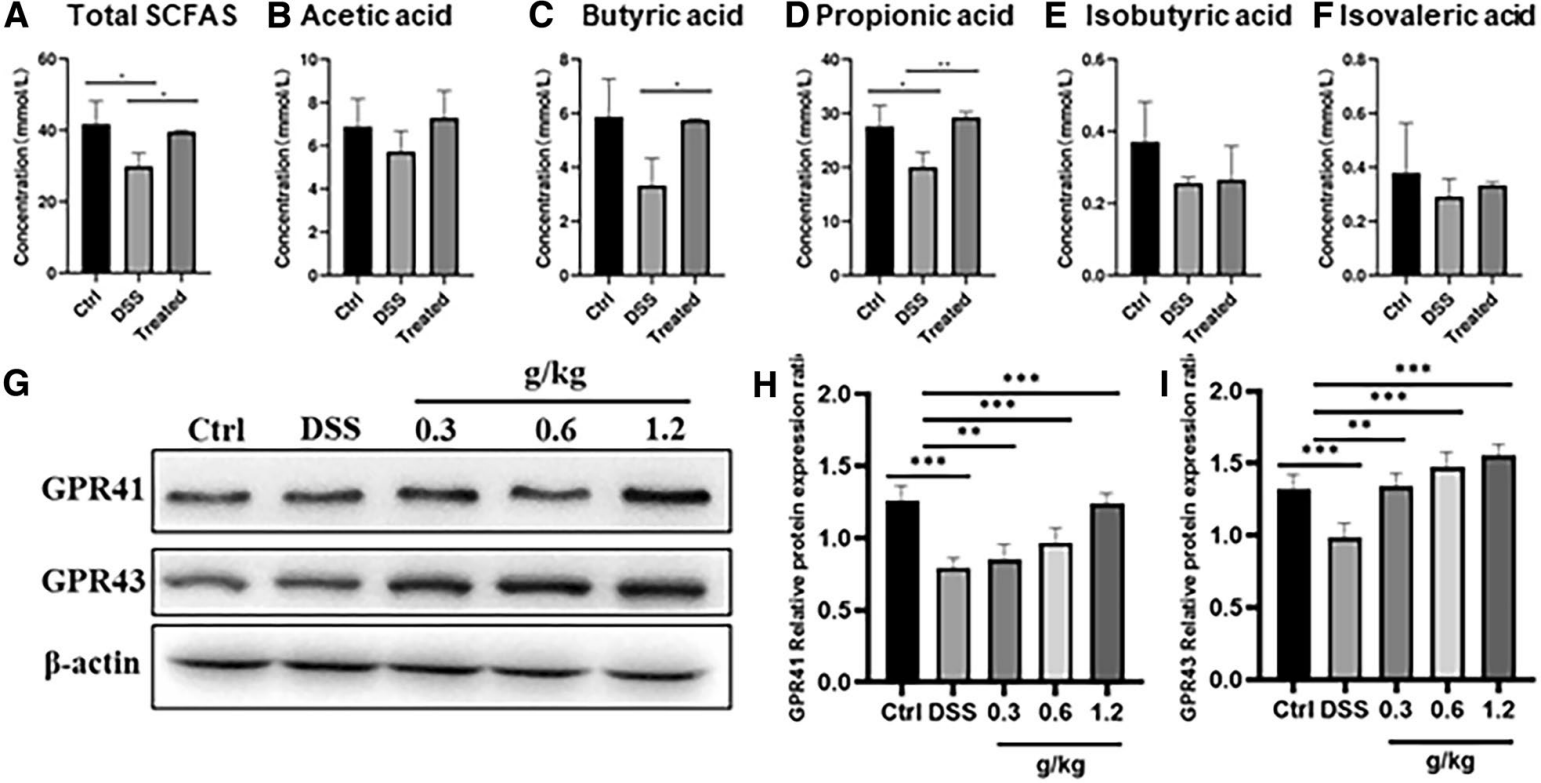
XYKJP promoted the secretion of SCFAs and activated GPR41/GPR43 proteins. **A** The statistical results of total SCFAs. **B** The statistical results of acetic acid. **C** The statistical results of propionic acid. **D** The statistical results of butyric acid. **E** The statistical results of isobutyric acid. **F** The statistical results of isovaleric acid. **G** The representative images of western blotting for GPR41 and GPR43. **H** The expressional changes of GPR41 proteins. **I** The expressional changes of GPR43 proteins. Data were expressed as ± SEM (n = 3), *P < 0.05, **P < 0.01, ***P < 0.001

SCFAs are the main metabolites of intestinal bacteria and are considered mediators of the intestinal microbiota in the regulation of intestinal immune function. In our study, we compared two groups: the DSS group, which was induced with dextran sulfate sodium to simulate a condition of intestinal inflammation, and the control group, which did not receive this induction. We found that the total SCFA content was lower in the DSS group than in the control group. However, XYKJP treatment significantly reversed this downregulation of total SCFAs (Fig. 4A) and markedly increased propionic acid (Fig. 4C) and butyric acid (Fig. 4D). GPR41/43 are SCFA receptors which have multiple functions in inflammation and oxidative stress [17], thus, we examined their expression levels. The results showed that GPR41/43 protein levels significantly decreased in the DSS group, whereas the decreased levels were inhibited by XYKJP (Fig. 4G–I). These results suggest that XYKJP promotes the repair of intestinal homeostasis by regulating the production of SCFAs and activating GPR41/43 proteins.

### The effect of XYKJP on oxidative stress in colitis tissues

**Fig. 5:**
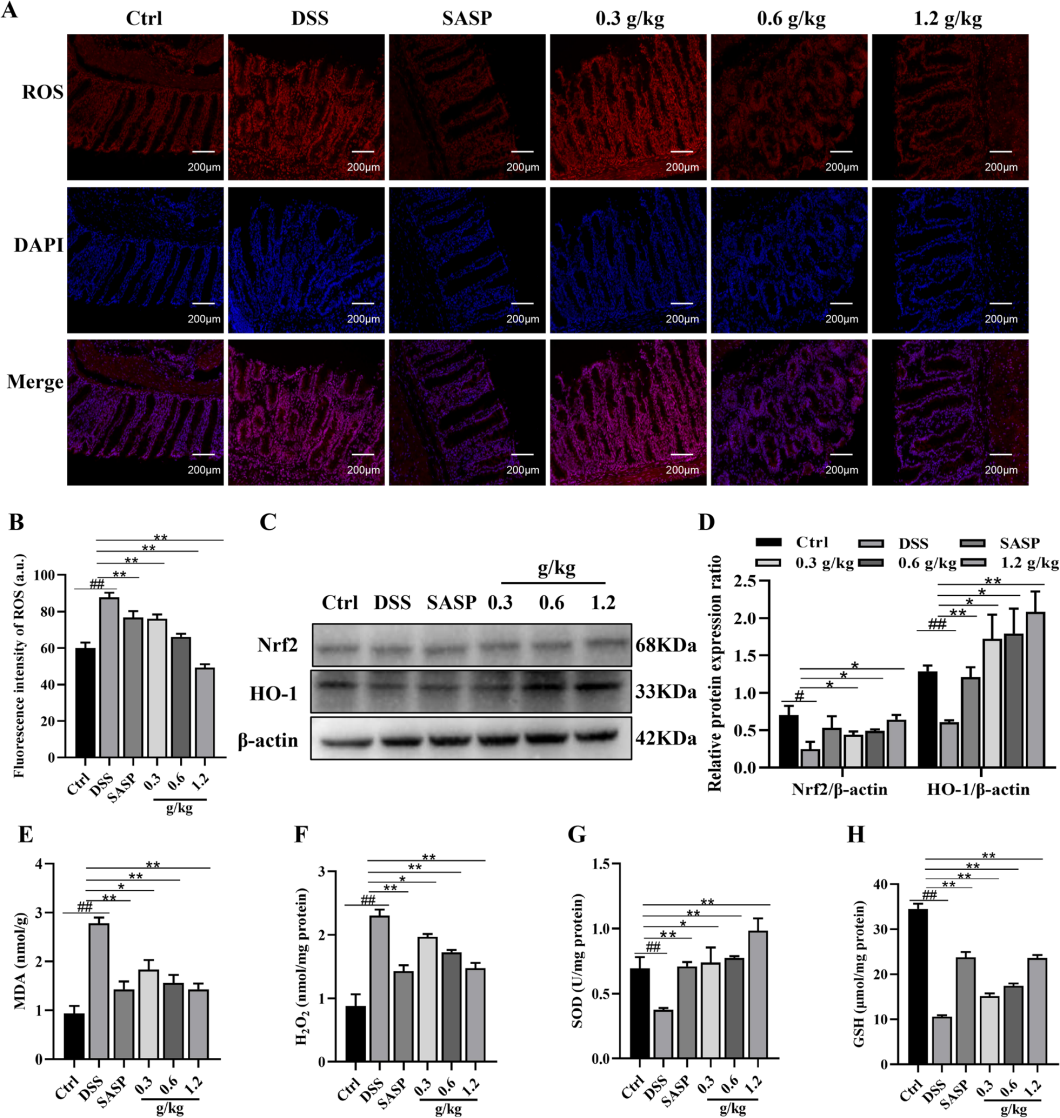
XYKJP ameliorated oxidative stress and regulated colon tissues' Nrf2/HO-1 signaling pathway. **A** The levels of ROS were measured using an IF assay with DAPI staining under a fluorescence microscope. **B** The statistical results of the semiquantitative image of ROS levels. **C** The representative western blotting images for Nrf2 and HO-1 proteins. **D** The expressional changes statistical results of Nrf2 and HO-1 proteins. **E**–**H** The statistical results of changes in the levels of MDA, H2O2, SOD, and GSH content, respectively. Data were expressed as ± SEM (n = 3), *P < 0.05, **P < 0.01, ***P < 0.001

Reactive oxygen species (ROS) production, a significant factor in oxidative stress imbalance and tissue damage, is a key pathological manifestation of colitis [18]. This prompted us to investigate the effects of XYKJP on oxidative stress in colon tissues. We found that the ROS level, significantly increased compared to the control group, could be effectively downregulated by XYKJP, with this effect becoming more pronounced with increasing XYKJP concentration (Fig. 5A, B). The DSS group showed decreased Nrf2 and HO-1 expression, which was significantly upregulated by XYKJP treatment (Fig. 5C, D). ELISA results showed a significant increase in the expression of superoxide dismutase (SOD) and glutathione (GSH), and a reduction in malondialdehyde (MDA) and H2O2 levels due to XYKJP treatment (Fig. 5E, H). These results, which highlight the key points of our study, provide a strong foundation for further research and potential clinical applications, instilling confidence in the robustness of our findings.

### XYKJP inhibited inflammation by regulating inflammatory cytokines and JAK2/STAT3 signaling pathways

**Fig. 6:**
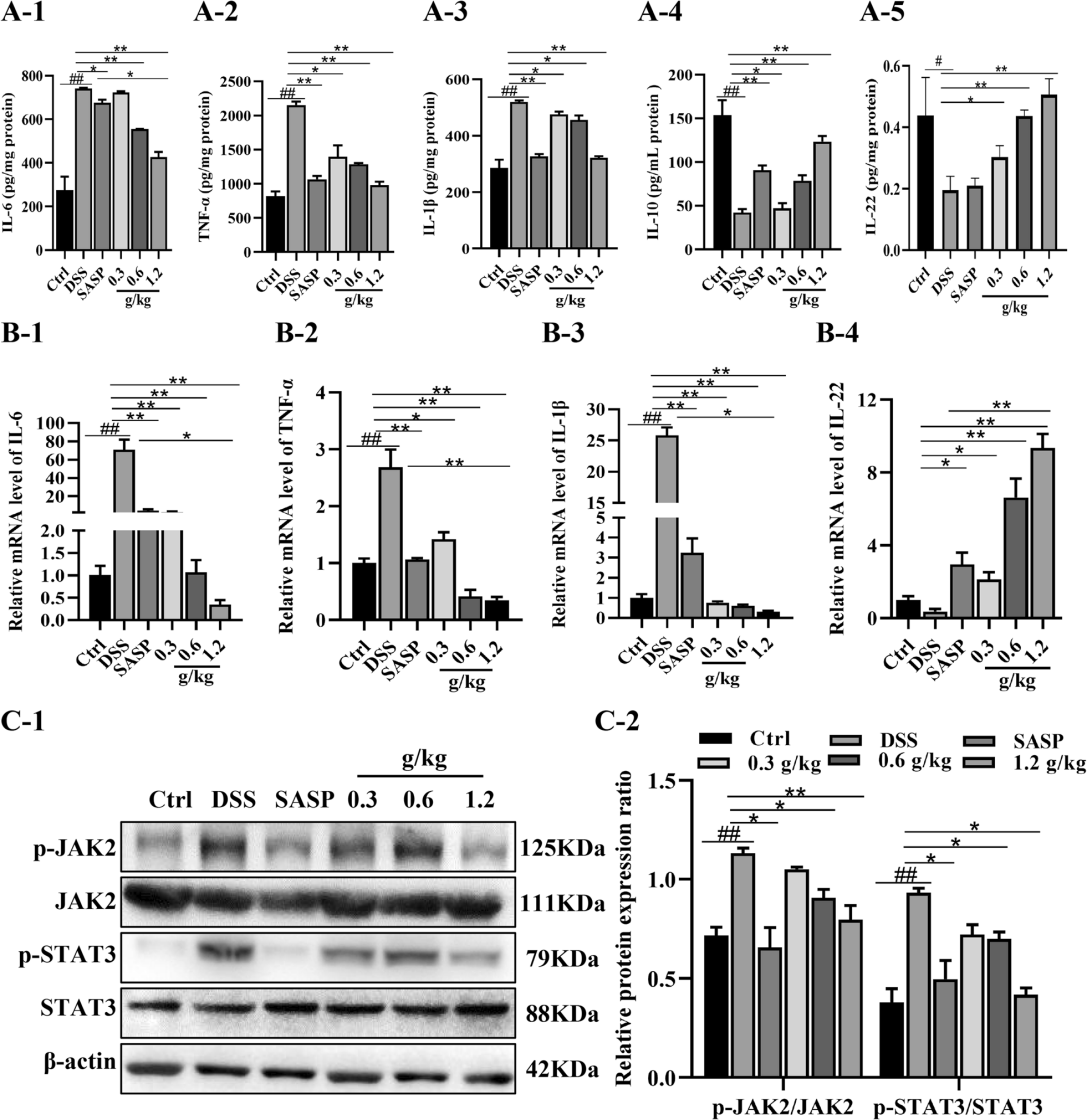
XYKJP regulated the inflammatory cytokine levels in DSS-induced colitis tissues. **A-1** to **A-5** The ELISA statistical results of IL-6, TNF-α, IL-1β, and IL-10, IL- 22. **B-1** to **B-4** The mRNA statistical results of IL-6, TNF-α, IL-1β, and IL-22. **C-1** The representative images of western blotting of p-JAK2, JAK2, p-STAT3, and STAT3 protein were normalized to β-actin. **C-2** The expressional changes statistical results of p-JAK2/JAK2, p-STAT3/ STAT3 protein by Image J software. Data were expressed as ± SEM (n = 3), *P < 0.05, **P < 0.01, ***P < 0.001

Inflammation, a crucial pathological response to colitis, was the focus of our investigation. We estimated the effect of XYKJP on inflammatory cytokines in colitis tissues and found that XYKJP treatment led to a reduction in the levels of IL-1β, TNF-α, and IL-6, and a significant improvement in the level of IL-10 and IL-22, as demonstrated by ELISA (Fig. 6A). Furthermore, our qRT-PCR results indicated that XYKJP inhibited IL-6, TNF-α, and IL-1β, and markedly increased the IL-22 expression (Fig. 6B). In the pathogenesis of colitis, IL-6 and IL-22 are inflammation-associated markers that correlate with the activation of the JAK and STAT3 pathways. Our western blot analysis revealed that XYKJP significantly suppressed JAK2 and STAT3 phosphorylation (Fig. 6C), suggesting that XYKJP's potential therapeutic effects on colitis may be mediated through the inhibition of the JAK2/STAT3 pathway.

### JAK2 inhibitor AG490 enhanced the XYKJP-related therapeutic effects of colitis

**Fig. 7:**
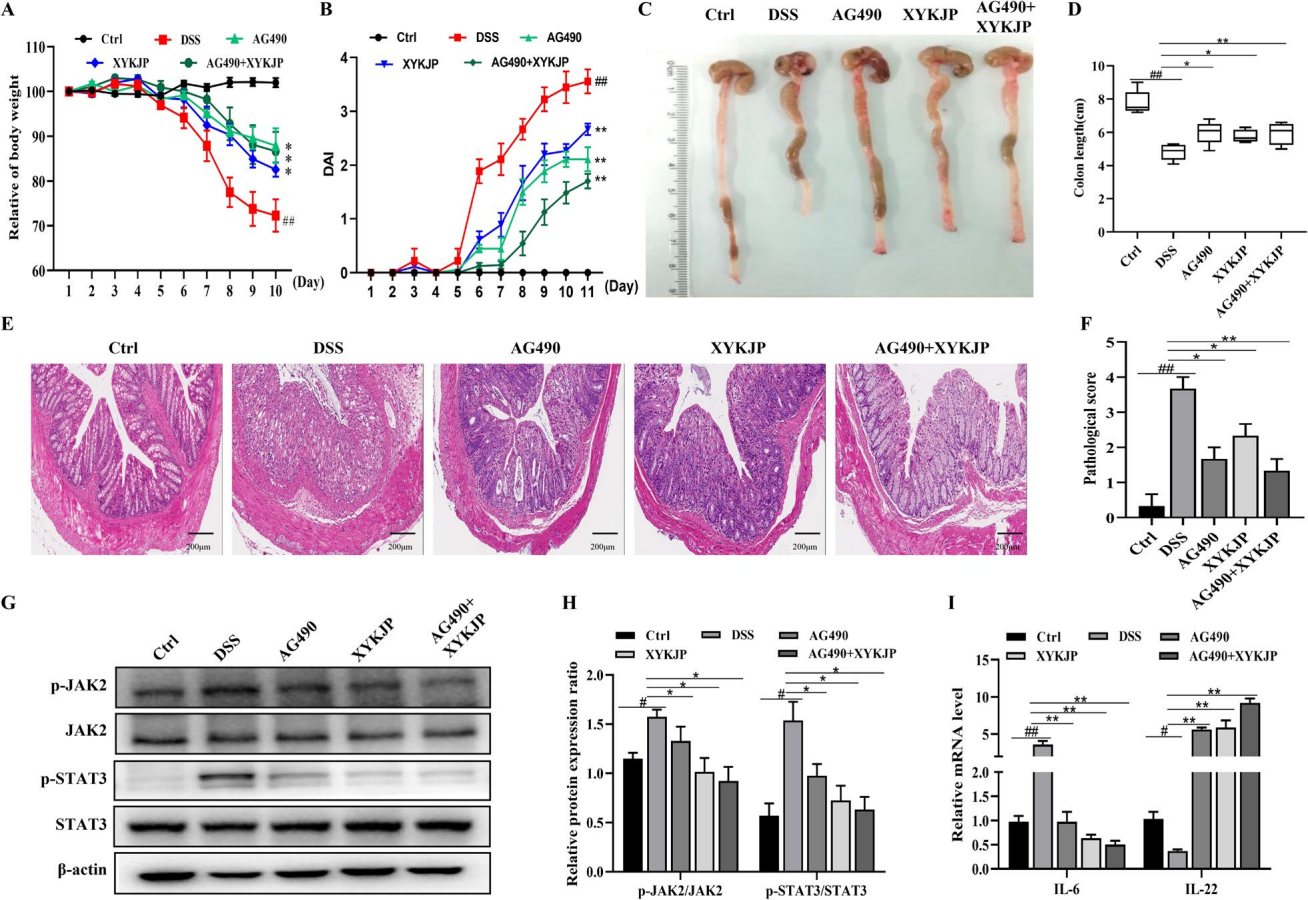
XYKJP may ameliorate DSS-induced colitis mainly by inhibiting the JAK2/STAT3 pathway in DSS-induced colitis tissues. **A** The daily changes of each group's mice body weight in 10 days. **B** The DAI scores of each group's mice were assessed daily for 10 days. **C**–**D** The result of each group's mice colon length was measured and then calculated. **E**–**F** The results of typical H&E pictures (scale bars = 200 μm) were obtained for pathologic evaluation. **G** The representative images of western blotting of p-JAK2, JAK2, p-STAT3, and STAT3 protein were normalized to β-actin. **H** The expressional changes statistical results of p-JAK2/JAK2, p-STAT3/ STAT3 protein by Image J software. **I** The statistical results of changes of mRNA for IL-6 and IL-22 by qRT-PCR. Data were expressed as ± SEM (n = 3), *P < 0.05, **P < 0.01, ***P < 0.001

Our exploration of the JAK2-specific inhibitor AG490 and its potential relationship with the protective effects of XYKJP has led to intriguing findings [19]. Compared to the DSS group, we observed a reversal of weight loss, but increased DAI following treatment with XYKJP and AG490 (Fig. 7A, B). The colon length was also considerably increased colon length by XYKJP and AG490 (Fig. 7C, D). H&E staining results further demonstrated that both XYKJP and AG490 treatment groups were able to reduce inflammatory infiltration and goblet cell loss at the colon site, and even restore the mucosal structure of the colon (Fig. 7E, F). Moreover, XYKJP treatment decreased p-JAK2 and p-STAT3 expression, in addition, XYKJP treatment decreased mRNA expression for IL-6 and increased IL-22 mRNA expression, which was significantly enhanced by AG490 (Fig. 7H, I). These results hint at the potential of XYKJP in inhibiting the JAK2/STAT3 pathway to protect the colon tissues, opening up new avenues for further research and potential clinical applications.

### XYKJP promoted intestinal mucosal repair by promoting tight junction (TJ) protein expression

**Fig. 8:**
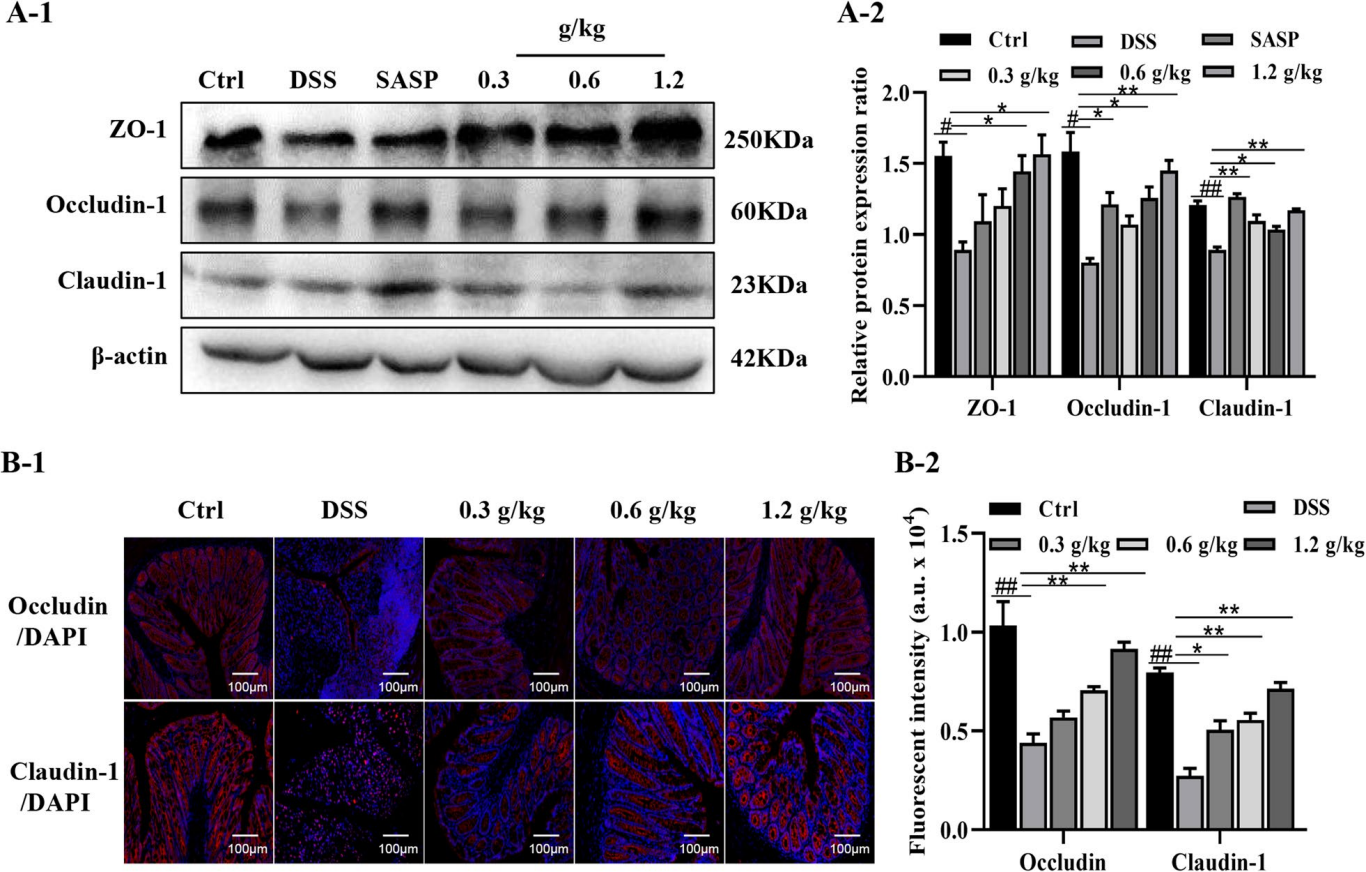
XYKJP improved the gut barrier disruption in colon tissues. **A-1** to **A-2** The representative images of ZO-1, occludin-1, and claudin-1 protein and the corresponding expression measured by image J software. **B-1** to **B-2** The expression of occludin-1 and claudin-1 in colon tissues was detected by IF. Data were expressed as ± SEM (n = 3), *P < 0.05, **P < 0.01, ***P < 0.001

The mucus layer of the intestinal epithelium, a crucial defense mechanism against pathogens, is primarily composed of TJ proteins, including zonula occluden-1 (ZO-1), occludin-1, and claudin-1. Our research, as depicted in Fig. 8A, revealed that the DSS group exhibited a reduced expression of ZO-1, occludin-1, and claudin-1, while XYKJP treatment increased the expression of these proteins in a dose-dependent manner, a significant finding in the field of gastroenterology and immunology. Immunofluorescence (IF) is a cellular imaging technique used to detect and localize proteins. Fluorescently labeled antibodies are conjugated to the detected protein molecules to form immunocomplexes that emit fluorescence. The location and expression of the target protein at the cellular or tissue level are observed by fluorescence microscopy. As in Fig. 8B, IF staining further supported these findings, which showed that occludin-1 and claudin-1 proteins were absent in colitis tissues but were localized in normal intestinal epithelial cell membranes after XYKJP treatment, suggesting that XYKJP can promote the repair of intestinal barrier function by increasing the expression of TJ proteins. Therefore, XYKJP promoted the recovery of colonic mucosal barrier function in mice with colitis by upregulating TJ protein expression.

### Correlation analysis among colitis, oxidative stress, inflammation, intestinal flora, and SCFAs

**Fig. 9:**
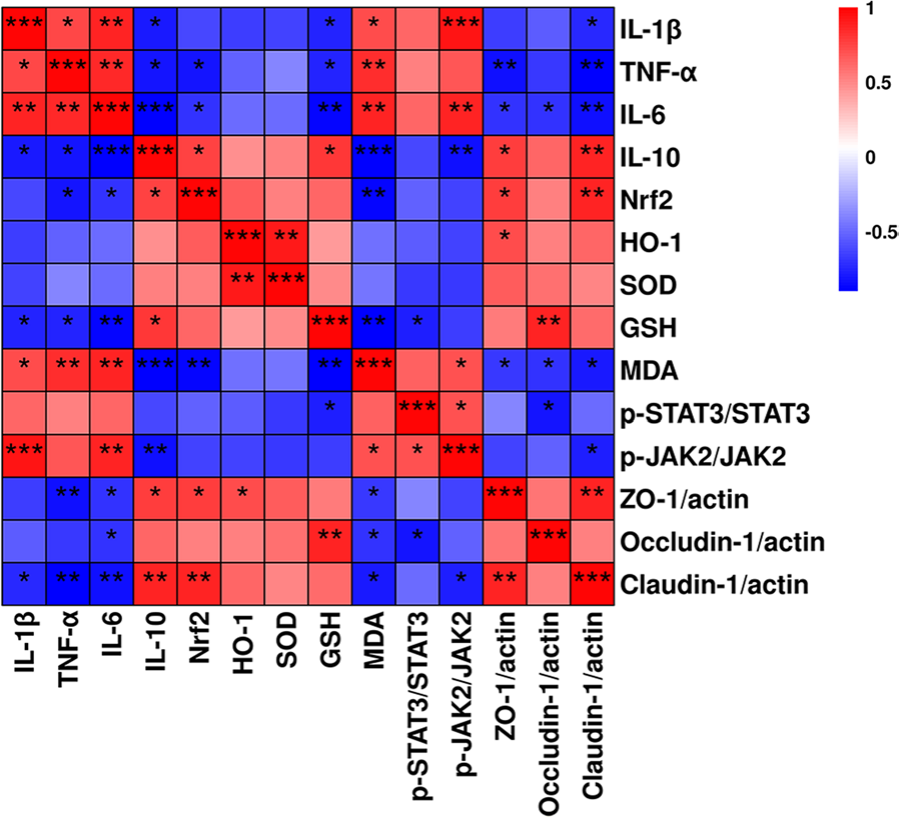
The heat map of Pearson's correlation coefficients among colitis, oxidative stress, inflammation, and intestinal flora and SCFAs. The color scale on the right indicated the correlation of samples, ranging from red (positive correlation) to light blue (negative correlation)

The correlations between Nrf2 and HO-1 expressions and various markers such as IL-10, SOD, GSH, acetic acid, and butyric acid levels, ZO-1, Occludin-1, and Claudin-1 expression, as well as their negative correlations with MDA IL-1β, and TNF-α levels (Fig. 9), present a complex web of relationships. Similarly, the positive correlations of p-STAT3/STAT3 and p-JAK2/JAK2 expression with IL-1β, TNF-α, and MDA levels, and their negative correlations with IL-10, acetic acid, butyric acid, GSH, and SOD, and the expressions of occludin-1, claudin-1, Nrf2, and HO-1, add to this intricate network.

## Discussion

Colitis is a significant public health challenge globally, necessitating the swift development of novel and potent drugs. XYKJP, a TCM preparation comprising Huang Qin, Diyu, and Shiliupi from the Wuhan University Renmin Hospital, has shown promise in treating intestinal disorders. In this study, we specifically investigated the role of XYKJP in treating colitis. Our results demonstrated that XYKJP effectively suppressed DSS-induced colitis in mice, as indicated by reduced weight loss, DAI score, colon length, and histological scores.

Recent studies have confirmed that intestinal flora imbalance plays an ineluctable or definite pathogenic role in the pathogenesis of colitis. Dysregulation of the gut microbiota and disruption of host symbiosis may be important factors in the progression of colitis [20]. Our results showed that *Akkermansia*, *Ruminococc-accac*_UCG_014, *Prevotellaceae*_Ga6A1_group, and *Ruminococca* cea_UCG_005, which are considered favorable for the production of SCFAs, were relatively enriched upon XYKJP treatment. SCFAs are one of the principal metabolites of the intestinal bacterial flora and are thought to be involved in several aspects of the host, including metabolic and immune processes. The Nrf2/HO-1 pathway and gut barrier-related genes in the intestine interact with SCFAs [21]. SCFAs can activate G protein-coupled receptors (GPRs) in intestinal epithelial cells, causing a series of downstream responses, including the regulation of various immune cell activities and participation in the regulation of the host immune system. XYKJP significantly increased the SCFA content, suggesting a potential breakthrough in the treatment of colitis, notably propionic and butyric acids. Butyrate acid and propanoic acid, key components of XYKJP, play a crucial role in reducing colonic mucosal injury and decreasing IL-1β, TNF-α, and IL-6 in the colitis mouse model. Furthermore, butyric acid can directly induce the expression of TJ proteins in epithelial tissues, thereby enhancing the intestinal mucosal barrier function [22]. Propionic acid in the gut has systemic anti-inflammatory effects [23]. GPR41/43/109a are the main receptors for propionic and butyric acids. Shao et al. reported that SCFA exerts anti-inflammatory and antioxidant functions through specific receptors such as GPR41, GPR43 [24]. XYKJP significantly increased the expression of GPR41/43. This knowledge about the role of SCFAs and GPR receptors in the therapeutic effects of XYKJP is crucial for understanding how XYKJP relieves the symptoms of colitis by modulating intestinal flora dysbiosis, improving beneficial bacterial abundance, increasing the concentration of SCFAs, and activating GPR41 /43 receptors.

The pathology of colitis is a complex process, and our research has shed light on a crucial aspect of it. We have found that it is accompanied by the formation of reactive oxygen species (ROS), which contribute to an imbalance in oxidative stress and organismal damage, causing an inflammatory response. MDA is a major degradation product produced when ROS attacks intracellular unsaturated fatty acids [25]. SOD and GSH are important antioxidants that balance redox reactions and reduce the damage caused by free radicals [26]. GA and BA inhibit ROS production and upregulate superoxide expression of SOD, CAT, and HO-1 [27]. Similarly, our results showed that XYKJP reduced MDA levels while increasing SOD and GSH levels, thus exhibiting antioxidative stress potential. These findings are of utmost importance for the protection of colitis tissues and for the future of colitis treatment.

The Nrf2/HO-1 pathway controls oxidative stress and maintains redox equilibrium [28]. Nrf2 is an important transcription factor in antioxidant stress and is mainly involved in the transcription of HO-1, which regulates intracellular ROS levels in response to various stimuli [29]. Hu et al. demonstrated that Huang Qin could activate Nrf2, critical for protecting cells from oxidative stress, inflammation, and apoptosis [10]. Similarly, our results confirmed that XYKJP upregulated the expression of Nrf2 and HO-1 to reduce oxidative stress. In addition, Nrf2 activates the expression of TJ proteins in intestinal epithelial cells, such as ZO-1, thereby protecting the intestinal barrier [30]. In our study, correlation analysis showed that ZO-1, Occludin-1, and Claudin-1 expression was positively correlated with Nrf2 expression. Additionally, XYKJP increased ZO-1, Occludin-1, and Claudin-1 expression, suggesting that XYKJP exerted a protective effect against the intestinal barrier and mucosal barrier damage.

Excessive imbalance in inflammatory responses is a major pathogenic factor in colitis. As Jang et al. reported, Huang qin could decrease the secretions of IL-1β, IL-6, and ROS and act as an immunomodulatory agent [31]. Consistent with this finding, XYKJP significantly modulated the overall levels of these factors in an anti-inflammatory manner, sparking interest in the potential of XYKJP to exert an anti-inflammatory effect by modulating the secretion of inflammatory cytokines.

IL-6/JAK2/STAT3 signaling is a vital axis involved in cell homeostasis and inflammatory signal amplification in various diseases, such as colitis [32]. DSS-induced colitis was alleviated by Gegen Qinlian decoction, which was mediated by the suppression of IL-6/JAK2/STAT3 signaling. The main components of XYKJP, BA, GA, and EA, are not just names, but they are the key players in reducing the phosphorylation of STAT3 and JAK2, and inhibiting STAT3 nuclear transfer [33, 34]. Our research also confirms that XYKJP inhibits IL-6 expression and phosphorylation of JAK2 and STAT3, thereby reducing the inflammatory response to colitis.

**Fig. 10:**
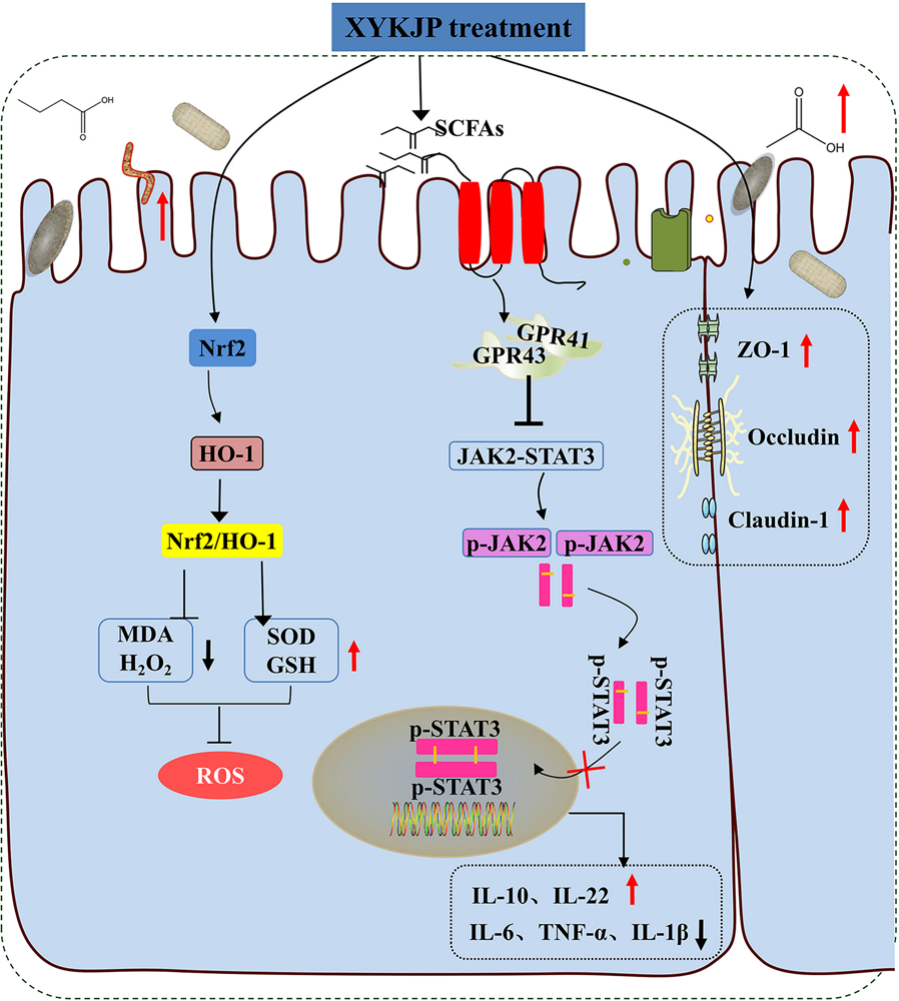
The possible mechanistic routes to XYKJP for the treatment of colitis

As shown in Fig. 10, XYKJP inhibited the synthesis of reactive oxygen species (ROS), hydrogen peroxide (H2O2, and malondialdehyde (MDA), promoted the synthesis of superoxide dismutase (SOD) and glutathione (GSH), and exerted antioxidant activity by triggering the Nrf2/HO-1 signaling pathway. By inhibiting the JAK2/STAT3 pathway, XYKJP exerted anti-inflammatory effects by regulating the secretion of IL-6, TNF-α, IL-1β, IL-10, and IL-22. XYKJP also enhanced the expression of TJ-related proteins, including ZO-1, Occludin-1, and Claudin-1, which are crucial for maintaining the integrity of the intestinal barrier function. Simultaneously, XYKJP can remodel the intestinal flora and promote the synthesis of SCFAs, including propionic and butyric acids. Based on this mechanism, XYKJP effectively ameliorated the pathological symptoms of colitis.

## Conclusion

In summary, our results showed that XYKJP effectively alleviated the symptoms of DSS-induced colitis in mice. Specifically, XYKJP modulated the composition of the intestinal flora, increased the content of SCFAs, especially propionic acid and butyric acid, and activated the expression of GPR41/43, a specific receptor for these acids, in model mice. This inhibits the activation of the JAK2/STAT3 pathway, which in turn regulates the secretion of inflammatory factors to exert anti-inflammatory effects and activates the Nrf2/HO-1 pathway to exert antioxidant effects. In addition, XYKJP increased the expression of tightly linked proteins and promoted the repair of intestinal barrier function. These results provide definitive evidence that further research in this area could lead to significant advancements in the treatment of colitis. XYKJP can treat colitis and suggests that it is a potential candidate for the treatment of colitis.

## Experimental section

### Materials

Dextran sulfate sodium (DSS; MW: 40,000) and metaphosphoric acid were purchased from Aladdin Technology (Shanghai, China), providing a diverse range of materials for our study. The myeloperoxidase (MPO) detection kit was purchased from Jiancheng Biotechnology Co., Ltd. (Nanjing, China). Mouse ELISA kits of interleukin (IL)-10, tumor necrosis factor (TNF)-α, IL-6 and IL-1β were obtained from Neobioscience (Shenzhen, China). Neutral-buffered paraformaldehyde was purchased from Wuhan Service Bio (Wuhan, China). An enhanced bicinchoninic acid (BCA) protein assay kit, NP-40 lysis buffer, protease and phosphatase inhibitor cocktails for mammalian cells and tissue extracts, and assay kits for superoxide dismutase (SOD), water-soluble tetrazolium-8, glutathione (GSH), and malondialdehyde (MDA) were obtained from Beyotime (Shanghai, China). Occludin-1, claudin-1, Zonula Occludens (ZO-1), signal transducer and activator of transcription 3 (STAT3), p-STAT3, nuclear factor erythroid 2-related Factor 2 (Nrf2), heme oxygenase (HO-1), Janus kinase 2 (JAK2), p-JAK2, GPR41/43 and β-actin were purchased from ABclonal Technology Co., Ltd. (Wuhan, China). A microplate reader (Spark 20 M Sunrise) was purchased from Tecan Trading (Shanghai, China). The electrophoresis apparatus, mini-transfer tank, and electrophoresis power supply were procured from Liuyi Biotechnology (Beijing, China).

Huangqin (Radix Scutellariae, the dried root of *Scutellaria baicalensis Georg*i) voucher specimens (No. SC-2019028), Shiliupi (Garden Burnet Root, dried root of *Sanguisorba officinalis* L.) voucher specimens (No. SC-2019016) and diyu (Pomegranate Rind, the dried rind of *Punica granatum* L.) voucher specimens (No. SC-2019020) were deposited in the Herbarium of Medical Plants, College of Pharmacy, South-Central Minzu University. XYKJP (pharmaceutical batch number: Z20180126) was provided by Renmin Hospital of Wuhan University. We used Sulfasalazine (SASP, Shanghai Sine Tianping Pharmaceutical Co., Ltd. Shanghai, China) enteric-coated tablets as positive controls, a choice that underscores the robustness of our study's methodology.

For the animal experiment, the XYKJP sample solution was prepared as follows: 0.3, 0.6, and 1.2 g/kg of XYKJP powder were dissolved in 20 mL of saline.

### Preparation of XYKJP

XYKJP is composed of Huangqin, Shiliupi, and Diyu. Huangqin, Shiliupi, and Diyu were weighed in a ratio of 3:2:2 and were extracted twice with six volumes of 60% ethanol for 1 h. The filtrate was concentrated, and the extract powder was obtained by spray drying. The dry extract powder was evenly mixed with crosslinked povidone, sodium carboxymethyl starch, and microcrystalline cellulose, and then a suitable amount of 60% ethanol solution was applied to make particles and dried at 50 °C, then pressed into tablets with a final drug dose of 30 mg/100 mg.

XYKJP is composed of Huangqin, Shiliupi, and Diyu. These ingredients were weighed in a ratio of 3:2:2 and were subjected to a thorough extraction process. They were extracted twice with six volumes of 60% ethanol for 1 h, ensuring the maximum extraction of beneficial compounds. The filtrate was concentrated, and the extract powder was obtained by spray drying. The dry extract powder was then mixed with crosslinked povidone, sodium carboxymethyl starch, and microcrystalline cellulose, and a suitable amount of 60% ethanol solution was applied to make particles and dried at 50 °C, then pressed into tablets with a final drug dose of 30 mg/100 mg. This thorough extraction process guarantees the efficacy of the product.

### LC–MS analysis of XYKJP components

The UHPLC system (Ultimate 3000 UPLC, Dionex) connected to a Q Exactive /MS (Thermo Scientific, Bremen, Germany) was selected to analyze the compound composition of XYKJP as the LC–MS platform. The main active ingredients were separated by using an Accucore^™^ aQ C18column (100 × 2.1 mm, 2.6 μm) with a constant flow of 0.2 mL/min. The column temperature was 25 °C. The injection volume was 5 μL. The mobile phases were solvent (A): Acetonitrile, B: 0.1% aqueous solution of formic acid. The gradient elution process was meticulously designed and executed: 95–90% (0–3 min), 90–83% A (3–9 min), 83–80% A (9–11 min), 80–72% A (11-14 min), 72–70% A (14–16 min), 70–65% A (16–19 min), 65–63% A (19–21 min), 63–45% (21–29 min), 45–37% (29–33 min), 37–5% (33–43 min). The samples were analyzed by a Q-Exactive mass spectrometer in an ESI ± ms ion source. The capillary temperature was 300 °C, the sheath gas was 30 Arb, the auxiliary gas was 10 Arb, the max spray current was 100.00 µA, and the probe heater temp was 320 °C.

### Animal experiments

All ethical procedures complied with the requirements of the Ethics Committee of South-Central Minzu University and followed the Guide for the Care and Use of Experimental Animals (Licence No. SYXK (Hubei) 2016–0089). C57BL/6 mice (SPF, 6–8 w), a commonly used model for this type of research due to their genetic stability and susceptibility to certain diseases, were supplied by the Liaoning Provincial Laboratory Animal Resource Center (Licence No: SCXK (Liaoning) 2020-01) and fed in an SPF animal laboratory.

The 60 mice were meticulously and randomly divided into 6 groups (n = 10), each serving a specific purpose: Ctrl group, DSS group (DSS), DSS + 0.3 g/kg XYKJP intervention group (L-XYKJP), DSS + 0.6 g/kg XYKJP intervention group (M-XYKJP), DSS + 1.2 g/kg XYKJP intervention group (H-XYKJP), and DSS + 0.2 g/kg SASP group (SASP). In the JAK2-STAT3 pathway inhibitor experiments, 50 mice were similarly and randomly divided into five groups (n = 10): Ctrl, DSS, DSS + H-XYKJP, DSS + AG490 (5 mg/kg, intraperitoneal), DSS + H-XYKJP + AG490 (5 mg/kg).

All mice were carefully acclimatized and fed for 7 days before the experiments, ensuring their comfort and well-being. On day 8, all mice, except the control group, were fed 3.0% DSS in drinking water for 1 week. XYKJP and SASP dissolved in saline were orally administered to mice in the corresponding groups for 10 consecutive days. The control and DSS groups were treated with saline (0.1 mL/10 g).

### Disease activity index (DAI) score and spleen index

After modeling and intervention in each group, the mice were observed and the DAI (including stool bleeding, stool consistency, and weight loss) was evaluated (Table 1). All mice were euthanized on day 18 with the utmost care and respect for ethical considerations, and the entire colon was extracted and measured for length. The spleen was collected and weighed to calculate the spleen index. All other samples were stored at −80 °C for subsequent evaluation.

**Table 1:** Evaluation of DAI scores

DAI score	Weight loss (%)	Stool consistency	Occult/gross bleeding
0	None	None	None
1	1–5	Soft	–
2	5–10	Loose stool	Hemoccult
3	10–15	–	–
4	> 15	diarrhea	Gross bleeding

### Histopathological assay

The isolated colon tissues were fixed with 4% paraformaldehyde and then placed in paraffin and cut into 4-μm-thick slices, then stained with hematoxylin and eosin (H&E). Colonic histopathologic changes were scored according to previous studies [35].

### Reactive oxygen species (ROS) measurement

ROS levels were detected by immunofluorescence analysis in a thorough and systematic manner. The process began with staining colon tissue sections using a ROS kit, followed by the incubation of the probes (dichlorodihydrofluorescein diacetate, DCFH-DA, 10 μM) in the dark at 37 °C for 30 min. This was then followed by three washes with high-dextrose dulbecco's modified eagle's medium (DMEM). The results, a product of our meticulous protocol, were observed using a fluorescence microscope (ECLIPSE Ti2, Nikon, Japan), and the fluorescence intensity (MFI) of ROS was assessed using ImageJ [36].

### Measurement of myeloperoxidase (MPO) activity

An MPO kit was used to determine MPO activity in the colon tissue. Briefly, appropriate colonic tissue was weighed and added to the prepared reagent at a ratio of 1:19. A 5% tissue homogenate was harvested, and the MPO activity was quantified. The OD density was measured at 460 nm. MPO activity was calculated using the following equation:

MPO (U/g colon tissues) = (OD value of the sample–OD value of the control)/11.3 × sample volume (g).

### Quantification of superoxide dismutase (SOD), malondialdehyde (MDA), and glutathione (GSH) levels

Colon tissue was homogenized on ice with 5 mL of 5% trichloroacetic acid (TCA) per gram. The homogenate was then centrifuged at 4 °C for 15 min at 1000 g, and the supernatant was collected for a comprehensive biochemical analysis.The levels of SOD, MDA and GSH were meticulously determined according to the manufacturer's instructions, leaving no stone unturned in the pursuit of accurate results.

### Cytokine measurements in colon tissue

To explore the effects of XYKJP's anti-inflammatory properties, the contents of tumor necrosis factor-α (TNF-α), interleukin 6 (IL-6), interferon-γ (IFN-γ), interleukin 10 (IL-10), and interleukin 1 beta (IL-1β) were measured by enzyme-linked immunosorbent assay (ELISA) kits respectively.

### Quantitative real-time PCR (qRT-PCR) analysis

The gene expression of inflammatory factors IFN-γ, IL-6, interleukin 22 (IL-22), IL-1β, and TNF-α was analyzed using qRT-PCR. Total RNA was extracted using the TRIzol reagent, followed by cDNA amplification. The primers used for the target genes are listed in Table 2. Finally, qRT-PCR (Applied Biosystems, Singapore) was performed, with the highly reliable β-actin serving as the reference gene, based on the 2(-ΔΔCt) method [37].

**Table 2:** List of primer sequences for RT-qPCR

Gene	Forward primer (5'–3')	Reverse primer (5'–3')
*β-actin*	GACCTGTACGCCAACACAGT	CTCAGGAGGAGCAATGATCT
*IL-6*	CCGCTATGAAGTTCCTCTC	GGTATCCTCTGTGAAGTCTC
*TNF-α*	AACTCCAGGCGGTGCCTATG	TCCAGCTGCTCCTCCACTTG
*IL-1β*	AGCTTCAGGCAGGCAGTATC	TCATCTCGGAGCCTGTAGTG
*IFN-γ*	ATGAACGCTACACACTGCATC	CCATCCTTTTGCCAGTTCCTC
*IL-22*	CATGCAGGAGGTGGTACCTT	CAGACGCAAGCATTTCTCAG

### Western blotting analysis

Proteins were enzymatically extracted using NP-40 lysis buffer containing a cocktail of protease and phosphatase inhibitors (50:1). The proteins were separated by 10% SDS-PAGE, a thorough process, and then transferred into a polyvinylidene difluoride (PVDF) membrane for 1.5 h. The membranes were then blocked with 5% skim milk and incubated overnight at 4 °C with primary antibodies (Table 3). Subsequently, the PVDF membranes were incubated with a secondary antibody for 1 h. The target proteins were identified using ECL reagents and analyzed using the ImageJ software (NIH Image, USA).

**Table 3:** Anti-body used for western blot experiment

Premary	Company	Cat	Dilution
occludin-1	ABclonal	A2601	1:1000
ZO-1	ABclonal	A0659	1:1000
claudin-1	ABclonal	A2196	1:800
p-STAT3	ABclonal	AP0705	1:1000
STAT3	ABclonal	A11216	1:1000
p-JAK2	ABclonal	AP0917	1:1000
JAK2	ABclonal	A11497	1:1000
Nrf2	ABclonal	A1244	1:1000
HO-1	ABclonal	A19062	1:1000
GPR41(FFAR3)	Sigma-Aldrich	MABN898	1:100
GPR43(FFAR2)	Sigma-Aldrich	ABC299	1:100
β-actin	ABclonal	AC026	1:200000

### 16S rRNA sequencing

To assess the regulatory effects of XYKJP on the intestinal flora, we performed 16S rRNA gene sequencing. The bacterial DNA in the colon contents was extracted using a DNA Kit (MN NucleoSpin 96 Soi), which was used to amplify the V4 region.16S rRNA gene V4 sequencing was conducted using an Illumina NovaSeq 6000 platform (Beijing Baimaike Biotechnology Co., Ltd, Beijing, China). We then provided detailed information on library construction, bacterial abundance analysis, and most importantly, the functional prediction of fecal samples, which holds the key to our significant findings, in the Supplementary Methods.

### Determination of short-chain fatty acids (SCFAs)

To detect the levels of SCFAs, we homogenized 50 mg of fecal samples with 50 ml of 1% (V/V) aqueous methanol solution (containing 25.0% HPO3), shaken on a vortex shaker for 10 min, then centrifuged at 1.2 × 104 g at 4 °C for 10 min, then collected supernatant for subsequent analysis. SCFA content was meticulously analyzed using gas chromatography (GC). These conditions are described in the Supplementary Methods.

### Statistical analysis

All the data were expressed as the mean ± SEM. The results were evaluated using Student's t-test and one-way analysis of variance (ANOVA). ImageJ software was used to analyze the histological and fluorescent images. Bar graphs were generated using the Prism 8.0.2 software (GraphPad, La Jolla, CA, USA).

## Supplementary Information

Supplementary material 1.

## Data Availability

Data will be made available on request.

## References

[1] Le Berre C, Honap S, Peyrin-Biroulet L. Ulcerative colitis. Lancet. 2023;402(10401):571–84. 10.1016/S0140-6736(23)00966-2. 37573077 10.1016/S0140-6736(23)00966-2

[2] Graham DB, Xavier RJ. Pathway paradigms revealed from the genetics of inflammatory bowel disease. Nature. 2020;578(7796):527–39. 10.1038/s41586-020-2025-2. 32103191 10.1038/s41586-020-2025-2 PMC7871366

[3] Agrawal M, Allin KH, Mehandru S, Faith J, Jess T, Colombel J-F. The appendix and ulcerative colitis - an unsolved connection. Nat Rev Gastro Hepat. 2023;20(9):615–24. 10.1038/s41575-023-00774-3. 10.1038/s41575-023-00774-3 PMC10527463 37081213

[4] Sandborn WJ, Vermeire S, Peyrin-Biroulet L, Dubinsky MC, Panes J, Yarur A, et al. Etrasimod as induction and maintenance therapy for ulcerative colitis (ELEVATE): two randomised, double-blind, placebo-controlled, phase 3 studies. Lancet. 2023;401(10383):1159–71. 10.1016/S0140-6736(23)00061-2. 36871574 10.1016/S0140-6736(23)00061-2

[5] Lopetuso LR, Deleu S, Godny L, Petito V, Puca P, Facciotti F, et al. The first international Rome consensus conference on gut microbiota and faecal microbiota transplantation in inflammatory bowel disease. Gut. 2023;72(9):1642–50. 10.1136/gutjnl-2023-329948. 37339849 10.1136/gutjnl-2023-329948 PMC10423477

[6] Wu Q, Wu X, Wang M, Liu K, Li Y, Ruan X, et al. Therapeutic mechanism of baicalin in experimental colitis analyzed using network pharmacology and metabolomics. Drug Des Devel Ther. 2023;17:1007–24. 10.2147/DDDT.S399290. 37025160 10.2147/DDDT.S399290 PMC10072146

[7] Han D, Wu Y, Lu D, Pang J, Hu J, Zhang X, et al. Polyphenol-rich diet mediates interplay between macrophage-neutrophil and gut microbiota to alleviate intestinal inflammation. Cell Death Dis. 2023;14(10):656. 10.1038/s41419-023-06190-4. 37813835 10.1038/s41419-023-06190-4 PMC10562418

[8] He H, Qin Q, Xu F, Chen Y, Rao S, Wang C, et al. Oral polyphenol-armored nanomedicine for targeted modulation of gut microbiota-brain interactions in colitis. Sci Adv. 2023;9(21):eadf3887. 10.1126/sciadv.adf3887. 37235662 10.1126/sciadv.adf3887 PMC10219598

[9] Li M-Y, Wu Y-Z, Qiu J-G, Lei J-X, Li M-X, Xu N, et al. Huangqin decoction ameliorates ulcerative colitis by regulating fatty acid metabolism to mediate macrophage polarization via activating FFAR4-AMPK-PPARα pathway. J Ethnopharmacol. 2023;311:116430. 10.1016/j.jep.2023.116430. 36997133 10.1016/j.jep.2023.116430

[10] Zhai L, Peng J, Zhuang M, Chang Y-Y, Cheng KW, Ning Z-W, et al. Therapeutic effects and mechanisms of Zhen-Wu-Bu-Qi Decoction on dextran sulfate sodium-induced chronic colitis in mice assessed by multi-omics approaches. Phytomedicine. 2022;99:154001. 10.1016/j.phymed.2022.154001. 35240530 10.1016/j.phymed.2022.154001

[11] Kimura Y, Sumiyoshi M. Two hydroxyflavanones isolated from Scutellaria baicalensis roots prevent colitis-associated colon cancer in C57BL/6 J mice by inhibiting programmed cell death-1, interleukin 10, and thymocyte selection-associated high mobility group box proteins TOX/TOX2. Phytomedicine. 2022;100:154076. 10.1016/j.phymed.2022.154076. 35378414 10.1016/j.phymed.2022.154076

[12] Zhang W, Ou L, Peng C, Sang S, Feng Z, Zou Y, et al. Sanguisorba officinalis L. enhances the 5-fluorouracil sensitivity and overcomes chemoresistance in 5-fluorouracil-resistant colorectal cancer cells via Ras/MEK/ERK and PI3K/Akt pathways. Heliyon. 2023;9(6):e16798. 10.1016/j.heliyon.2023.e16798. 37484409 10.1016/j.heliyon.2023.e16798 PMC10360953

[13] Zhou P, Lai J, Li Y, Deng J, Zhao C, Huang Q, et al. Methyl gallate alleviates acute ulcerative colitis by modulating gut microbiota and inhibiting TLR4/NF-κB pathway. Int J Mol Sci. 2022;23(22):14024. 10.3390/ijms232214024. 36430509 10.3390/ijms232214024 PMC9697899

[14] Larsen IS, Jensen BAH, Bonazzi E, Choi BSY, Kristensen NN, Schmidt EGW, et al. Fungal lysozyme leverages the gut microbiota to curb DSS-induced colitis. Gut Microbes. 2021;13(1):1988836. 10.1080/19490976.2021.1988836. 34693864 10.1080/19490976.2021.1988836 PMC8547870

[15] Scaioli E, Belluzzi A, Ricciardiello L, Del Rio D, Rotondo E, Mena P, et al. Pomegranate juice to reduce fecal calprotectin levels in inflammatory bowel disease patients with a high risk of clinical relapse: study protocol for a randomized controlled trial. Trials. 2019;20(1):327. 10.1186/s13063-019-3321-8. 31171016 10.1186/s13063-019-3321-8 PMC6554985

[16] Haifer C, Paramsothy S, Kaakoush NO, Saikal A, Ghaly S, Yang T, et al. Lyophilised oral faecal microbiota transplantation for ulcerative colitis (LOTUS): a randomised, double-blind, placebo-controlled trial. Lancet Gastroenterol. 2022;7(2):141–51. 10.1016/S2468-1253(21)00400-3. 10.1016/S2468-1253(21)00400-3 34863330

[17] Lin Y, Lv Y, Mao Z, Chen X, Chen Y, Zhu B, et al. Polysaccharides from Tetrastigma Hemsleyanum Diels et Gilg ameliorated inflammatory bowel disease by rebuilding the intestinal mucosal barrier and inhibiting inflammation through the SCFA-GPR41/43 signaling pathway. Int J Biol Macromol. 2023;250:126167. 10.1016/j.ijbiomac.2023.126167. 37558022 10.1016/j.ijbiomac.2023.126167

[18] Hsu N-Y, Nayar S, Gettler K, Talware S, Giri M, Alter I, et al. NOX1 is essential for TNFα-induced intestinal epithelial ROS secretion and inhibits M cell signatures. Gut. 2023;72(4):654–62. 10.1136/gutjnl-2021-326305. 36191961 10.1136/gutjnl-2021-326305 PMC9998338

[19] Gan F, Lin Z, Tang J, Chen X, Huang K. Deoxynivalenol at no-observed adverse-effect levels aggravates DSS-induced colitis through the JAK2/STAT3 signaling pathway in mice. J Agric Food Chem. 2023;71(9):4144–52. 10.1021/acs.jafc.3c00252. 36847760 10.1021/acs.jafc.3c00252

[20] Maphetu N, Unuofin JO, Masuku NP, Olisah C, Lebelo SL. Medicinal uses, pharmacological activities, phytochemistry, and the molecular mechanisms of Punica granatum L. (pomegranate) plant extracts: a review. Biomed. 2022;153:113256. 10.1016/j.biopha.2022.113256. 10.1016/j.biopha.2022.113256 36076615

[21] Kim H, Banerjee N, Ivanov I, Pfent CM, Prudhomme KR, Bisson WH, et al. Comparison of anti-inflammatory mechanisms of mango (Mangifera Indica L.) and pomegranate (Punica Granatum L.) in a preclinical model of colitis. Mol Nutr Food Res. 2016;60(9):1912–23. 10.1002/mnfr.201501008. 27028006 10.1002/mnfr.201501008 PMC5026564

[22] Zhou D, Wu Y, Yan H, Shen T, Li S, Gong J, et al. Gallic acid ameliorates calcium oxalate crystal-induced renal injury via upregulation of Nrf2/HO-1 in the mouse model of stone formation. Phytomedicine. 2022;106:154429. 10.1016/j.phymed.2022.154429. 36099652 10.1016/j.phymed.2022.154429

[23] Lavelle A, Sokol H. Gut microbiota-derived metabolites as key actors in inflammatory bowel disease. Nat Rev Gastroenterol Hepatol. 2020;17(4):223–37. 10.1038/s41575-019-0258-z. 32076145 10.1038/s41575-019-0258-z

[24] Wan Y, Yang L, Jiang S, Qian D, Duan J. Excessive apoptosis in ulcerative colitis: crosstalk between apoptosis, ROS, ER stress, and intestinal homeostasis. Inflamm Bowel Dis. 2022;28(4):639–48. 10.1093/ibd/izab277. 34871402 10.1093/ibd/izab277

[25] Zhou S, Dai Q, Huang X, Jin A, Yang Y, Gong X, et al. STAT3 is critical for skeletal development and bone homeostasis by regulating osteogenesis. Nat Commun. 2021;12(1):6891. 10.1038/s41467-021-27273-w. 34824272 10.1038/s41467-021-27273-w PMC8616950

[26] Huang T, Liu Y, Zhang C. Pharmacokinetics and bioavailability enhancement of baicalin: a review. Eur J Drug Metab Pharmacokinet. 2019;44(2):159–68. 10.1007/s13318-018-0509-3. 30209794 10.1007/s13318-018-0509-3

[27] Liang J, Zhou Y, Cheng X, Chen J, Cao H, Guo X, et al. Baicalin attenuates H2O2-induced oxidative stress by regulating the AMPK/Nrf2 signaling pathway in IPEC-J2 cells. Int J Mol Sci. 2023;24(11):9435. 10.3390/ijms24119435. 37298392 10.3390/ijms24119435 PMC10253293

[28] Miao Y, Zheng Y, Geng Y, Yang L, Cao N, Dai Y, et al. The role of GLS1-mediated glutaminolysis/2-HG/H3K4me3 and GSH/ROS signals in Th17 responses counteracted by PPARγ agonists. Theranostics. 2021;11(9):4531–48. 10.7150/thno.54803. 33754076 10.7150/thno.54803 PMC7977454

[29] Suzuki T, Takahashi J, Yamamoto M. Molecular basis of the KEAP1-NRF2 signaling pathway. Mol Cells. 2023;46(3):133–41. 10.14348/molcells.2023.0028. 36994473 10.14348/molcells.2023.0028 PMC10070164

[30] Amirshahrokhi K, Imani M. Levetiracetam attenuates experimental ulcerative colitis through promoting Nrf2/HO-1 antioxidant and inhibiting NF-κB, proinflammatory cytokines and iNOS/NO pathways. Int Immunopharmacol. 2023;119:110165. 10.1016/j.intimp.2023.110165. 37068340 10.1016/j.intimp.2023.110165

[31] Jang JY, Im E, Kim ND. Therapeutic potential of bioactive components from Scutellaria baicalensis Georgi in inflammatory bowel disease and colorectal cancer: a review. Int J Mol Sci. 2023;24(3):1954. 10.3390/ijms24031954. 36768278 10.3390/ijms24031954 PMC9916177

[32] Elhefnawy EA, Zaki HF, El Maraghy NN, Ahmed KA, Abd El-Haleim EA. Genistein and/or sulfasalazine ameliorate acetic acid-induced ulcerative colitis in rats via modulating INF-γ/JAK1/STAT1/IRF-1, TLR-4/NF-κB/IL-6, and JAK2/STAT3/COX-2 crosstalk. Biochem Pharmacol. 2023;214:115673. 10.1016/j.bcp.2023.115673. 37414101 10.1016/j.bcp.2023.115673

[33] Zhou J, Li M, Chen Q, Li X, Chen L, Dong Z, et al. Programmable probiotics modulate inflammation and gut microbiota for inflammatory bowel disease treatment after effective oral delivery. Nat Commun. 2022;13(1):3432. 10.1038/s41467-022-31171-0. 35701435 10.1038/s41467-022-31171-0 PMC9198027

[34] Park WY, Song G, Park JY, Ahn KS, Kwak HJ, Park J, et al. Ellagic acid improves benign prostate hyperplasia by regulating androgen signaling and STAT3. Cell Death Dis. 2022;13(6):554. 10.1038/s41419-022-04995-3. 35715415 10.1038/s41419-022-04995-3 PMC9205887

[35] Fu Y-J, Xu B, Huang S-W, Luo X, Deng X-L, Luo S, et al. Baicalin prevents LPS-induced activation of TLR4/NF-κB p65 pathway and inflammation in mice via inhibiting the expression of CD14. Acta Pharmacol Sin. 2021;42(1):88–96. 10.1038/s41401-020-0411-9. 32457419 10.1038/s41401-020-0411-9 PMC7921675

[36] Hu Q, Zhang W, Wu Z, Tian X, Xiang J, Li L, et al. Baicalin and the liver-gut system: pharmacological bases explaining its therapeutic effects. Pharmacol Res. 2021;165:105444. 10.1016/j.phrs.2021.105444. 33493657 10.1016/j.phrs.2021.105444

[37] Li M-X, Li M-Y, Lei J-X, Wu Y-Z, Li Z-H, Chen L-M, et al. Huangqin decoction ameliorates DSS-induced ulcerative colitis: role of gut microbiota and amino acid metabolism, mTOR pathway and intestinal epithelial barrier. Phytomedicine. 2022;100:154052. 10.1016/j.phymed.2022.154052. 35344714 10.1016/j.phymed.2022.154052

