# Correction: Xiaoyankangjun tablet alleviates dextran sulfate sodium‑induced colitis in mice by regulating gut microbiota and JAK2/STAT3 pathway

**DOI:** 10.1007/s13659-025-00536-5

**Published:** 2025-08-13

**Authors:** Suqin Yang, Jingtao Huang, Wenjing Tan, Xiankun Xia, Dali Gan, Yalei Ren, Hanwen Su, Meixian Xiang

**Affiliations:** 1 School of Pharmaceutical Sciences, South-Central Minzu University, Wuhan, 430074 Hubei People’s Republic of China; 2 Department of Clinical Laboratory, Renmin Hospital of Wuhan University, Wuhan, 430060 Hubei People’s Republic of China

**Correction: Natural Products and Bioprospecting (2024) 14:44** 10.1007/s13659-024-00468-6

Following publication of the original article [1], the author reported a mis-placed WB panel in Fig. 4G and would like to update the figure from:
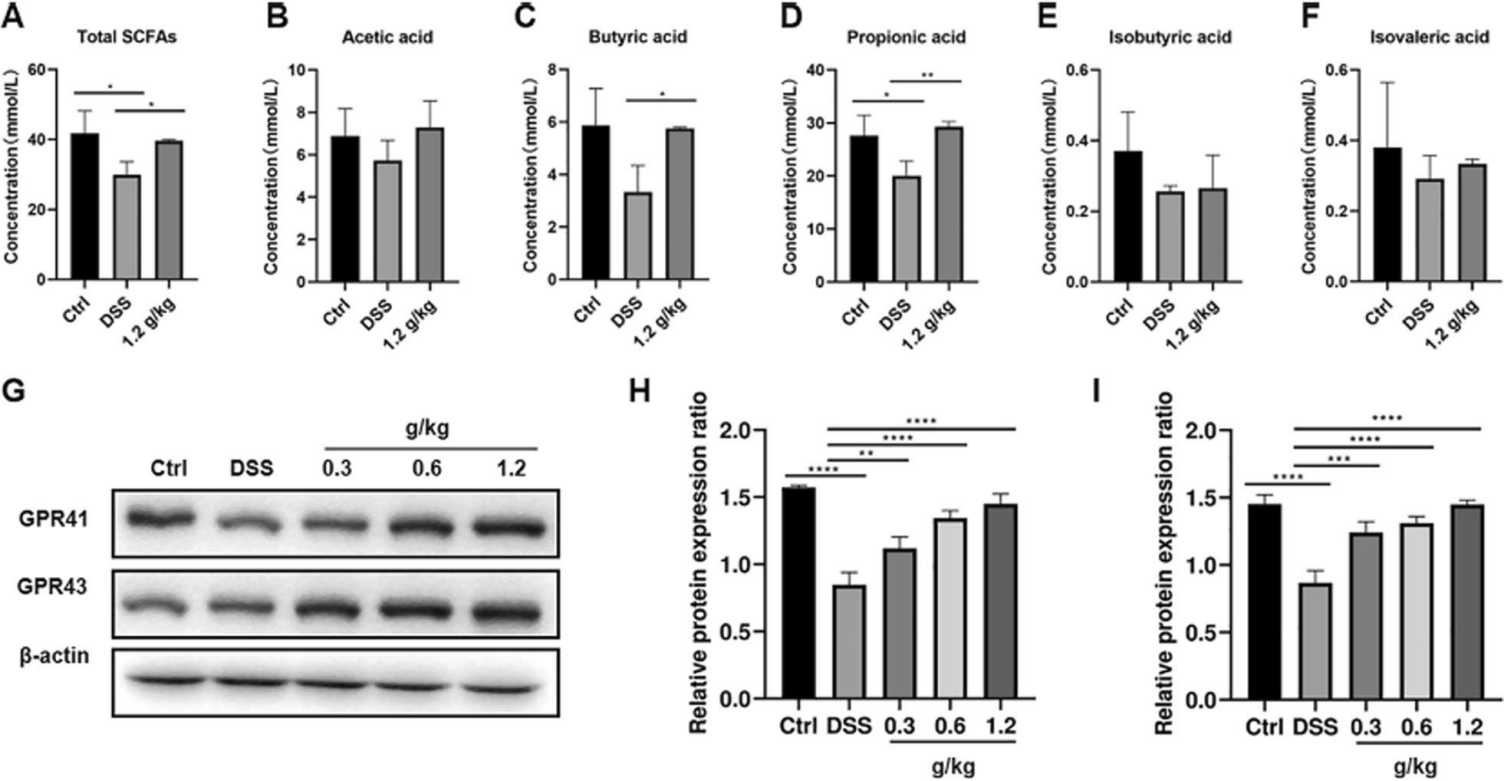


To:

See Fig. 4.

**Fig. 4:**
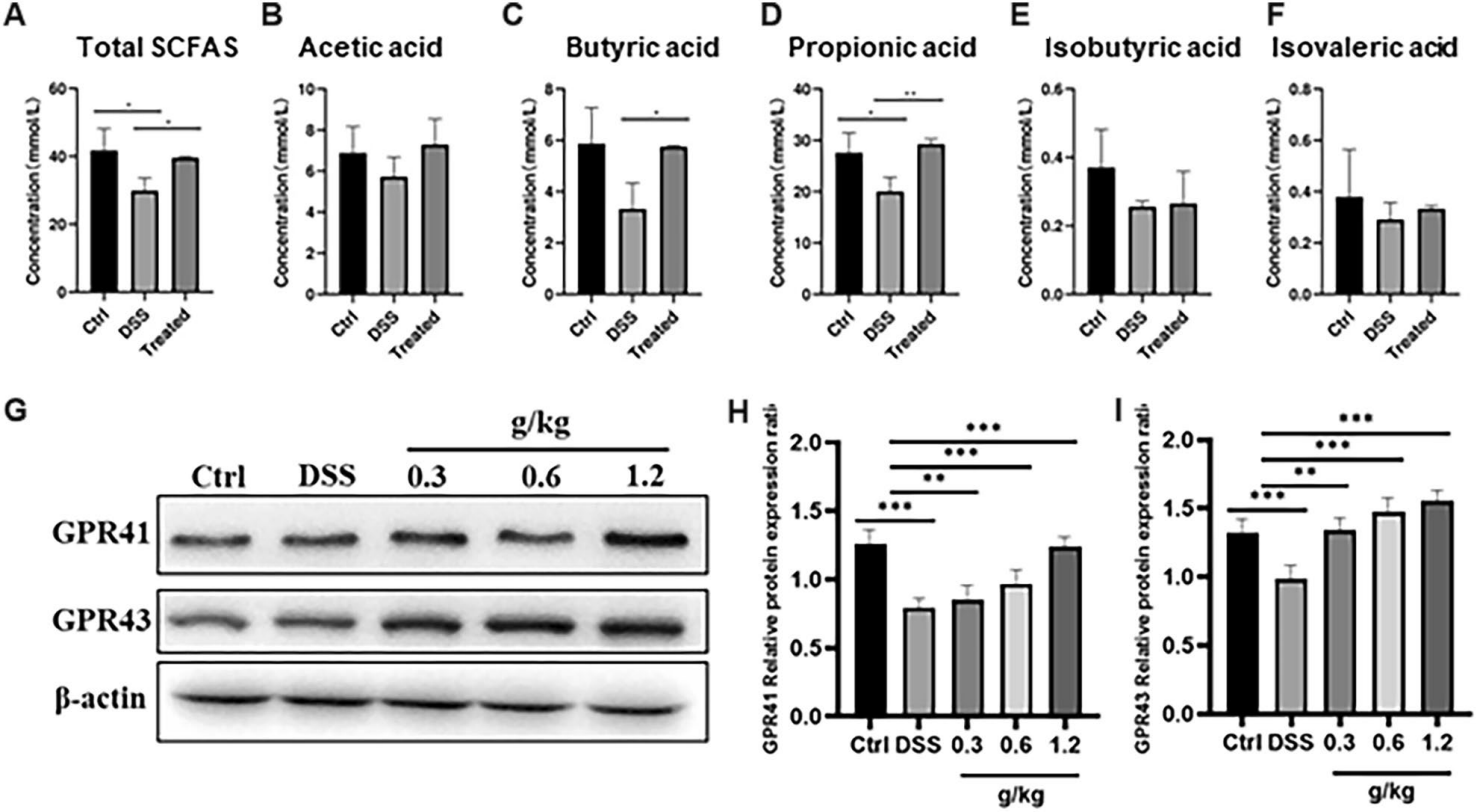
XYKJP promoted the secretion of SCFAs and activated GPR41/GPR43 proteins. **A** The statistical results of total SCFAs. **B** The statistical results of acetic acid. **C** The statistical results of propionic acid. **D** The statistical results of butyric acid. **E** The statistical results of isobutyric acid. **F** The statistical results of isovaleric acid. **G** The representative images of western blotting for GPR41 and GPR43. **H** The expressional changes of GPR41 proteins. **I** The expressional changes of GPR43 proteins. Data were expressed as ± SEM (n = 3), *P < 0.05, **P < 0.01, ***P < 0.001

The original article [1] has been updated.
