# Supplementary material for: Xiaoyankangjun tablet alleviates dextran sulfate sodium-induced colitis in mice by regulating gut microbiota and JAK2/STAT3 pathway
Source: Nat Prod Bioprospect. 2024 Aug 12;14(1):44. doi: 10.1007/s13659-024-00468-6 (PMC11319580; doi:10.1007/s13659-024-00468-6)
Supplement: Supplementary file 1 — Supplementary material 1. [file 13659_2024_468_MOESM1_ESM.docx]

# Supplementary data

## 16S rRNA Sequencing

The quality of the DNA samples was tested before use to construct libraries, and the primers 515F (5'-GTGYCAGCMGCCGCGGTAA-3') and 806 R2 (5'-GGACTACNVGGGTWT CTAAT-3') were to amplify the V4 region. PCR amplification products were purified using OMEGA DNA purification columns. 16S rRNA gene V4 sequencing was conducted with an Illumina NovaSeq 6000 platform (Beijing Baimaike Biotechnology Co., Ltd, Beijing, China). Each sample of raw data was spliced with FLASH software (version 1.2.11); low-quality reads were filtered out to the clean data (Xie et al., 2022). The consensus sequence was obtained by stitching (Trimmomatic, version 0.33), simultaneous removal of chimeras (UCHIME, version 8.1) for high-quality labeled sequences, clustered 97% of the clean labels into operable taxonomic units (OTUs) with USEARCH (version 10.0). The representative OTUs sequences were grouped using the Ribosomal Database Project (RDP) Classifier (version 2.2) at a confidence threshold of 0.8.

The α- diversity and β-diversity were analyzed with QIIME software (version 2.0). Moreover, the β-diversity was calculated as intersample distances using the unweighted unifrac algorithm to reveal differences between samples. The principal coordinates analysis (PCoA) plots were generated by R software (version 4.1.0). The significant difference between groups at the phylum and genus levels was analyzed with Metastats (http://metastats.cbcb. umd.edu/).

## Determination of short-chain fatty acids (SCFAs)

50 mg feces were collected and transferred into 2 mL centrifuge tubes, then added 50 µL of methanol for suspension, and the tube was shaken on a vortex shaker for 10 min, and then centrifuged at 1.2 ×104 g at 4 °C for 10 min, then transferred 100 μL supernatant into a new 2 mL centrifuge tube, mixed with 25% metaphosphoric acid (HPO3) at 5:1 ratio, and kept at 4 °C for overnight, then centrifuged at 1.2 ×104 g at 4 °C for 10 min, then collected supernatant for subsequent analysis.

The SCFAs content was analyzed by a gas chromatograph (TRACE 1300, Thermo Fisher Scientific, USA) with a TR-FAME column (60 m × 0.25 mm ID × 0.25 μm). The temperature was initially set to 75 °C, the temperature was maintained for 2 min when increased to 180 °C at 6 °C/min. The temperature of the detector was 230 °C, the sample volume was 1 μL, the carrier gas was nitrogen (N2), and the flow rate was 3 mL/min.


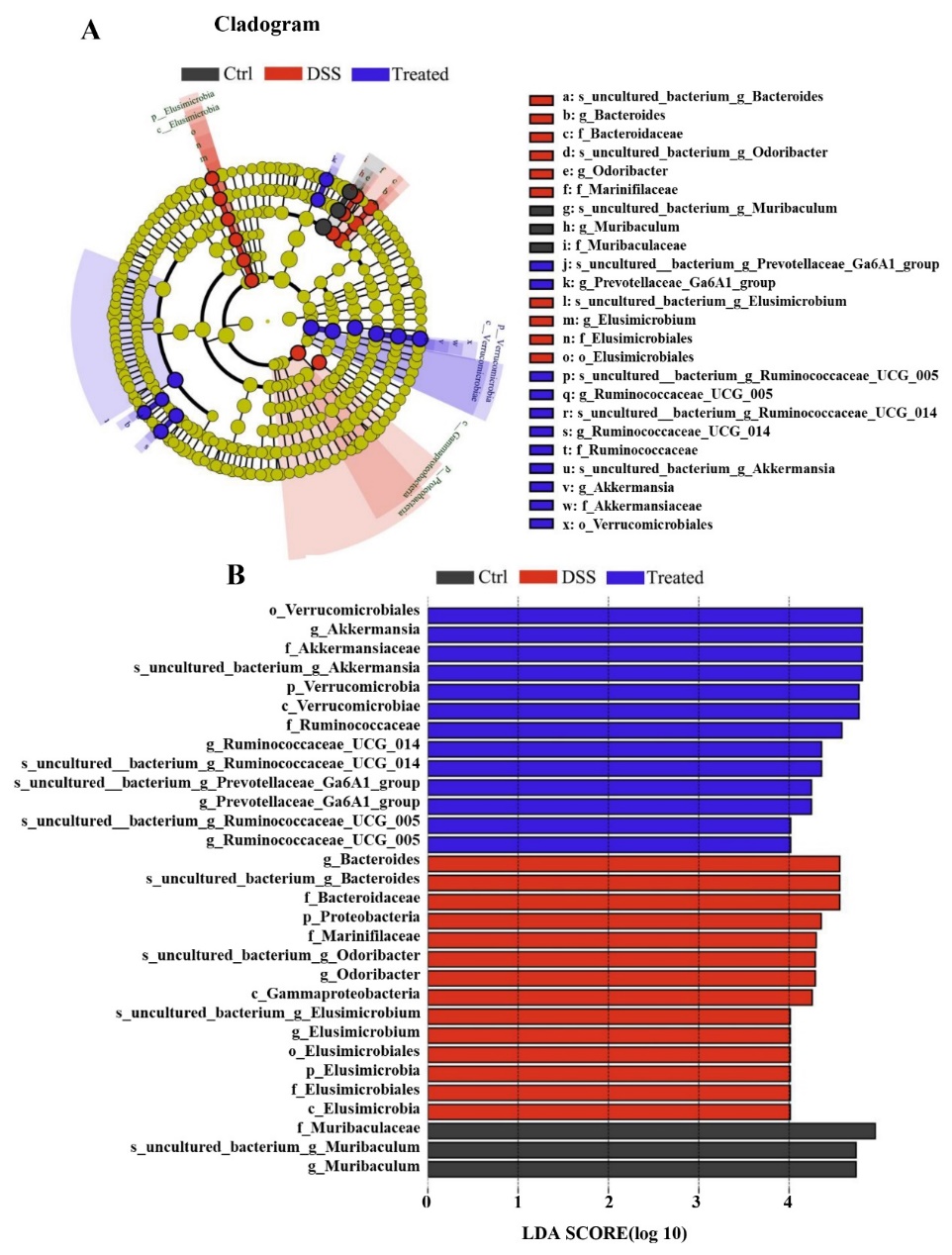


**Fig. S1. Identification of feature classification unit with the greatest difference among three experimental groups.** (A) Analysis of feature classification unit among three experimental groups by LEfSe analysis. The inner to outer circles indicated the level of bacteria from phylum to genus. The yellow dots represented bacteria that were not significantly different from each other, and the characteristic bacteria were colored according to their corresponding class color. (B) Feature classification unit using LDA with a threshold score > 4.0. Bar length of LDA represented the impact of characteristic taxa in individual groups.
